# Type of diet has no major influence on inflammatory response in a Saddleback pig model

**DOI:** 10.1038/s41598-025-92420-y

**Published:** 2025-03-11

**Authors:** Lisa Wahl, Susanne Rau, Christine A. Dawczynski, Stefan Lorkowski, Reiner Ulrich, Matthias Blüher, Ingrid Vervuert

**Affiliations:** 1 Institute of Animal Nutrition, Nutrition Diseases and Dietetics, Leipzig University, Leipzig, Germany; 2 Competence Cluster of Nutrition and Cardiovascular Health (nutriCARD), Halle-Jena-Leipzig, Jena, Germany; 3 Institute of Veterinary Pathology, Leipzig University, Leipzig, Germany; 4 Junior Research Group Nutritional Concepts, Institute of Nutritional Sciences, Friedrich Schiller University, Jena, Germany; 5 Institute of Nutritional Sciences, Friedrich Schiller University Jena, Jena, Germany; 6 Helmholtz Institute for Metabolic, Obesity and Vascular Research (HI-MAG) of the Helmholtz Zentrum München at Leipzig University and University Hospital Leipzig, Leipzig, Germany

**Keywords:** Inflammation, Obesity, Pectin, Inulin, Short-chain fatty acids, Nutritional supplements, Experimental models of disease, Translational research, Obesity, Obesity

## Abstract

Fermentable carbohydrates and resulting short-chain fatty acids (SCFAs) received attention via modifying potential on obesity-associated systemic low-grade inflammation. However, their effects on inflammation remain poorly understood. In this study, the anti-inflammatory properties of pectin or inulin supplementation were investigated in an atherogenic-fed pig obesity model. Pigs were divided into three atherogenic-fed groups with or without 5% pectin/inulin supplementation (AD, ADp, ADi, *n* = 10) and a conventional-fed group (CD, *n* = 10) for a 15-week feeding period. We demonstrated that faecal SCFA concentrations decreased and faecal pH increased in all groups over the feeding period (*P* < 0.05). SCFA concentrations were comparable between colon and faeces in all groups. Liver inflammatory-marker expressions were on average < 1 in all groups, except *TNF-α* (AD < CD and ADi; *P* < 0.01). Inflammatory-marker expressions in abdominal adipose tissue exceeded subcutaneous marker expressions in all groups. AD showed significantly lower *IL-1β* and *CD68* mRNA levels than CD (*P* < 0.03). Comparing the atherogenic diet groups, the *IL-1β* mRNA levels were higher in ADi versus AD and ADp (*P* = 0.02). Our data indicated that fermentable carbohydrates added to an atherogenic diet cannot resolve low-grade adipose tissue inflammatory associated with obesity.

**Supplementary Information:**

The online version contains supplementary material available at 10.1038/s41598-025-92420-y.

## Introduction

In humans, obesity remains one of the greatest public health concerns because of its increasing prevalence and comorbidities such as metabolic and cardiovascular diseases^1^.

With the progressive severity of obesity, the secretion of bioactive molecules from adipose tissue (adipokines) changes towards a proinflammatory pattern such that obesity is associated with systemic low-grade inflammation in humans^2–4^. Adipokines influence the regulation of satiety (e.g., leptin and vaspin), endothelial function (e.g., omentin and apelin), haemostasis (e.g., fibrinogen), blood pressure (e.g., angiotensinogen), insulin sensitivity (e.g., adiponectin) and adipogenesis (e.g., retinol-binding protein-4)^3,5^. Therefore, adipose tissue is considered one of the largest endocrine organs in the body^6^. In humans, abdominal adipose tissue has been identified as particularly high risk and closely linked to metabolic disease, whereas subcutaneous adipose tissue attributed protective effects^3,5,6^. Immigrated immune cells, such as macrophages, are also involved in the systemic inflammatory process in obesity and are attracted by adipokines (e.g., monocyte-chemotactic-protein-1, progranulin and macrophage-inflammatory-protein-1α)^3,6^. Therefore, proinflammatory adipokines such as interleukin-1β (IL-1β), interleukin-6 (IL-6) and tumour necrosis factor α (TNF-α) are secreted by adipocytes as well as by adipose tissue immune cells^3^. The effect of these inflammatory molecules has been suggested to be a molecular link between adipose tissue and the metabolic and cardiovascular complications of obesity in humans^3,6,7^. A considerable inflammatory reaction of abdominal adipose tissue in contrast to that of subcutaneous fat has also been observed in obesity models in pigs^8–10^.

Various strategies for treating obesity and its metabolic consequences, in addition to promoting weight reduction, have been investigated^11–13^. Dietary supplements have been proposed as a potential strategy to counteract comorbidities of obesity^14,15^. In particular, fermentable carbohydrates such as pectin and inulin have attracted great attention in the treatment of obesity comorbidities, as they may influence the microbiome and its fermentation products, including SCFAs^16,17^. SCFAs such as acetate, propionate and butyrate may have a suppressive effect on inflammatory marker expression^18^. The underlying mechanisms by which SCFAs affect inflammation in the context of obesity are not completely understood. However, various studies have shown that fermentable carbohydrates and the resulting SCFAs have beneficial effects at both the intestinal and systemic level^19^.

Fermentable carbohydrates and the resulting SCFAs promote the proliferation of commensal microbial bacteria and can thereby positively influence the intestinal mucosal structure^20^. This leads to reduced translocation of bacterial endotoxins such as lipopolysaccharides (LPS) and an attenuated activation of toll-like receptors on the luminal surface of the gut^21^. In addition, fermentable carbohydrates increase the endogenous, intestinal production of glucagon-like peptide 2, which enhances the barrier function of the gut by upregulating tight junction proteins in the mucosa^22^. SCFAs and butyrate in particular are used as energy sources by intestinal epithelial cells^23^. Some SCFAs enter the liver and are partly metabolized^19^. A small percentage of SCFAs are distributed in the peripheral vascular system, where anti-inflammatory effects on various cells have been postulated^24^. Only 5–10% of SCFAs are excreted in faeces^25^. SCFAs are natural inhibitors of histone deacetylases (HDACs) and thus influence various signalling cascades in immune cells^19,26^. HDACs can regulate the secretion of proinflammatory cytokines (e.g., IL-1β, IL-2, IL-3, IL-5, IL-12, IL-18, TNF-α and TNF-β) by influencing the mitogen-activated protein kinase pathways and by inhibiting the acetylation of nuclear factor-κB (NF-κB), which mediates transcription^18^. In this context, butyrate has the most potent inhibitory effect on NF-κB activity, followed by propionate and acetate^19,27^. Furthermore, SCFAs exert anti-inflammatory effects by inhibiting the recruitment and migration of immune cells (macrophages, neutrophil granulocytes and dendritic cells) and the differentiation of T and B lymphocytes through target cell receptors (free fatty acid receptors and G-protein-coupled receptors)^18^. Garland (2011) reported that SCFAs may also play a role in immune response regulation of adipocytes^28^.

Several studies have shown that inulin or oligofructose attenuates the proinflammatory effect of a high-fat diet by reducing the LPS concentration in serum^29,30^. In a study conducted by Macfarlane et al.. (2013) in humans, TNF-α levels were reduced in peripheral blood via an inulin-containing symbiotic^31^. In pigs, inulin supplementation led to downregulation of *TNF-α* mRNA levels in the large intestine^32^. Pectin has also been shown to have anti-inflammatory, immunostimulatory and antioxidant effects^33,34^. In a study conducted by Li et al. (2018), pectin supplementation in combination with a high-fat diet in mice lowered hepatic *TNF-α* mRNA levels^35^. In rats, *TNF-α* and *IL-6* mRNA levels were systemically decreased^36^. A reduced expression of proinflammatory cytokines (*IL-1β*, *TNF-α*) in the gut after LPS challenge was also observed in pigs fed a pectin-supplemented diet^37^.

Animal models of obesity are important for experimental research investigating disease mechanisms and therapeutic strategies^38,39^. In this context, diet-induced obesity models in rodents and pigs have provided important insights into the pathophysiology of obesity^40,41^. Pig models are preferred because of their similarity to humans in terms of their food spectrum, digestive system, cardiovascular anatomy and physiology^42–44^. An atherogenic diet-induced obesity model in Saddleback pigs has been previously described by Wahl et al.. 2022^45^.

The aim of the current study was to investigate selected markers of inflammation in a Saddleback pig model under an atherogenic diet supplemented with pectin or inulin. We hypothesized that pectin and inulin may reduce inflammatory responses in obese pigs mediated by the modulation of SCFAs.

## Results

### Health monitoring

All pigs in the groups ADi and CD were clinically healthy during the 15-week feeding period. In the AD group, three pigs (one pig twice) were treated with nonsteroidal anti-inflammatory drugs (Melosolute: 20 mg/mL meloxicam; CP-Pharma, Burgdorf, Germany) for five to seven days during the 15-week feeding period because of lameness resulting from joint inflammation. Two of the animals also developed fever and were additionally treated with antibiotics (Hostamox LA: 150 mg/mL amoxicillin; MSD, Rahway, NJ, USA) for three days. In the ADp group, two animals were treated with nonsteroidal anti-inflammatory drugs for three days each. One of the pigs was moderately lame due to an inflamed bursa, and the other pig was lame as a result of slipping on the stable gangway. To avoid any interference with the microflora or inflammatory response in the liver and adipose tissue, an interval of at least two weeks was maintained between the treatment and the collection of samples. All of the sick pigs were successfully treated and did not need to be excluded from the experiment at any of the sampling time points. Supporting information on this topic is provided in a supplementary file S1.

### Lipid parameters in serum increased significantly by the atherogenic diet

The serum parameters triglycerides (TG; t1 and t2: *P* ≤ 0.01), bile acids (BA; t1 and t2: *P* ≤ 0.02) and hepatic triglyceride lipase (LIPC; t1: *P* ≤ 0.02) presented significantly greater values in the atherogenic diet groups than in the CD group. A detailed evaluation of the serum parameters of liver and lipid metabolism [TG, BA, LIPC, cholesterol (CHOL), alkaline phosphatase (ALP), aspartate aminotransferase (AST), gamma-glutamyl transferase (GGT), lactate dehydrogenase (LDH) and amylase (AMYL)] is described in Wahl et al.. (2022)^45^.

### Faecal pH and dry matter increased independently of diet type

**Fig. 1:**
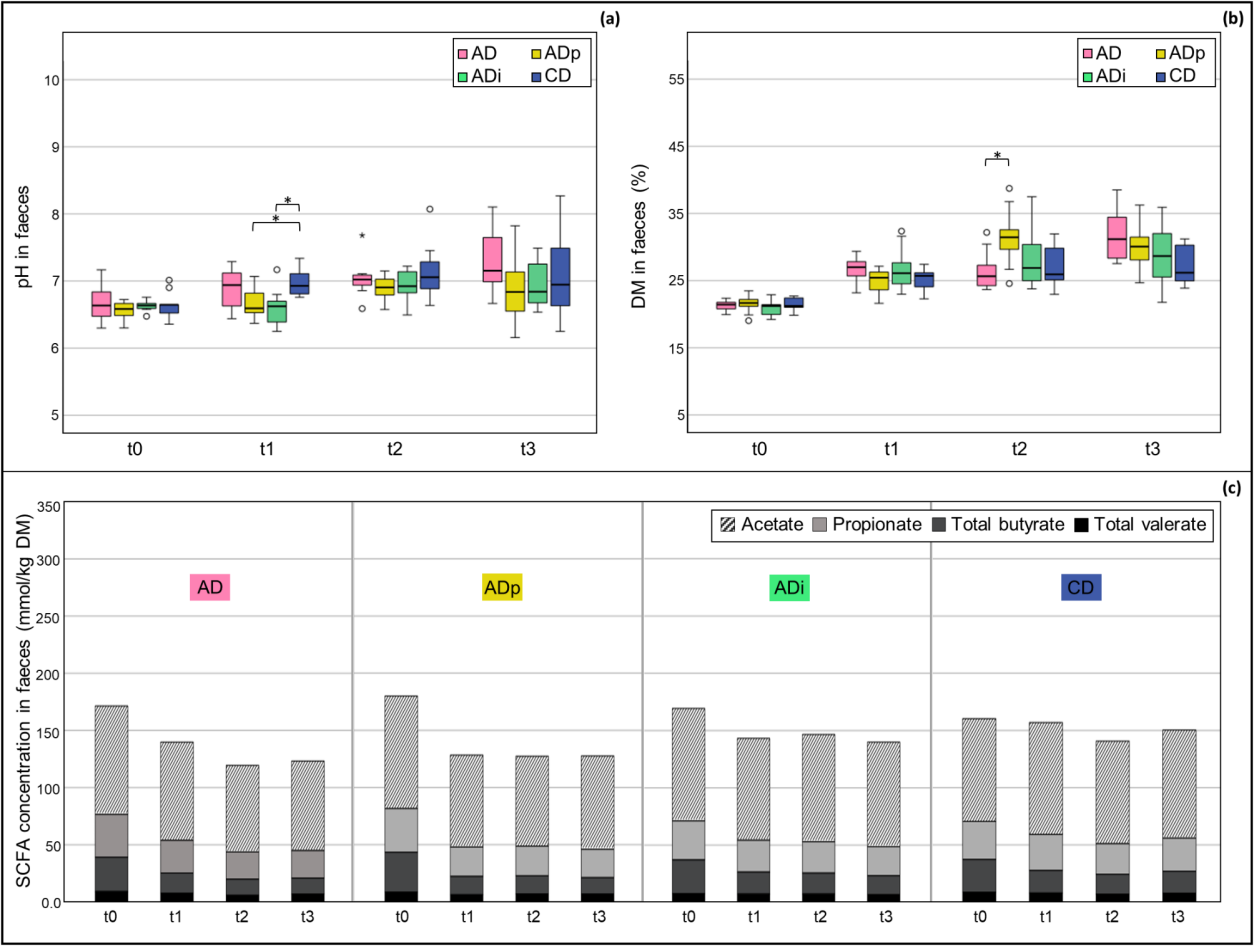
pH values, DM content (%) and SCFA concentrations (mmol/kg DM) in faeces. The data represent the time points before the feed change (t0) and at 1–3 months after the feed change (t1–t3) for all the feeding groups (AD, ADp, ADi and CD). *Asterisks indicate significant differences between the groups. Significant differences were identified by *P* values ≤ 0.05 via ANOVA with Games Howell test for pH values and the Kruskal–Wallis test with Bonferroni correction for DM content. DM, dry matter; SCFA, short-chain fatty acid; AD, group fed the atherogenic diet (*n* = 8–10); ADp, group fed the atherogenic diet + pectin (*n* = 9–10); ADi, group fed the atherogenic diet + inulin (*n* = 9–10); CD, group fed the conventional diet (*n* = 9–10).

An increase in the faecal pH and dry matter (DM) content was found in all the groups from t0 to t3 (Fig. 1a,b and Table 1 in Supplementary File S1). There were no significant differences in pH and DM content between the AD and CD group at any time (t0–t3). At t1, the pH was markedly higher in the CD group than in the ADp and ADi groups (*P* = 0.01), without significant differences between AD, ADp and ADi. At t2, the DM content of the AD group was significantly lower than that of the ADp group (*P* = 0.05).

### SCFA concentrations in faeces decreased during the feeding period, especially in pigs fed the atherogenic diet

The SCFA concentration in faeces decreased over time from t0–t3 in all groups (Fig. 1c and Table 1 in Supplementary File S1). A decrease was particularly observed in the AD, ADp and ADi groups, in which the feed was changed to an atherogenic diet after t0. No significant differences in SCFAs such as acetate, propionate, total butyrate and total valerate were found between the AD and CD groups at any time point (t0–t3), except before feed change (t0) with higher propionate levels in the AD group (*P* = 0.04). There were also no significant differences in SCFA levels between the AD, ADp and ADi groups at any time point (t0–t3). At t1, the faecal acetate and valerate levels were significantly lower in the ADp group than in the CD group (*P* ≤ 0.04).

### Highest chyme SCFA concentrations were found in the large intestine and SCFA levels were comparable between colon and faeces

**Fig. 2:**
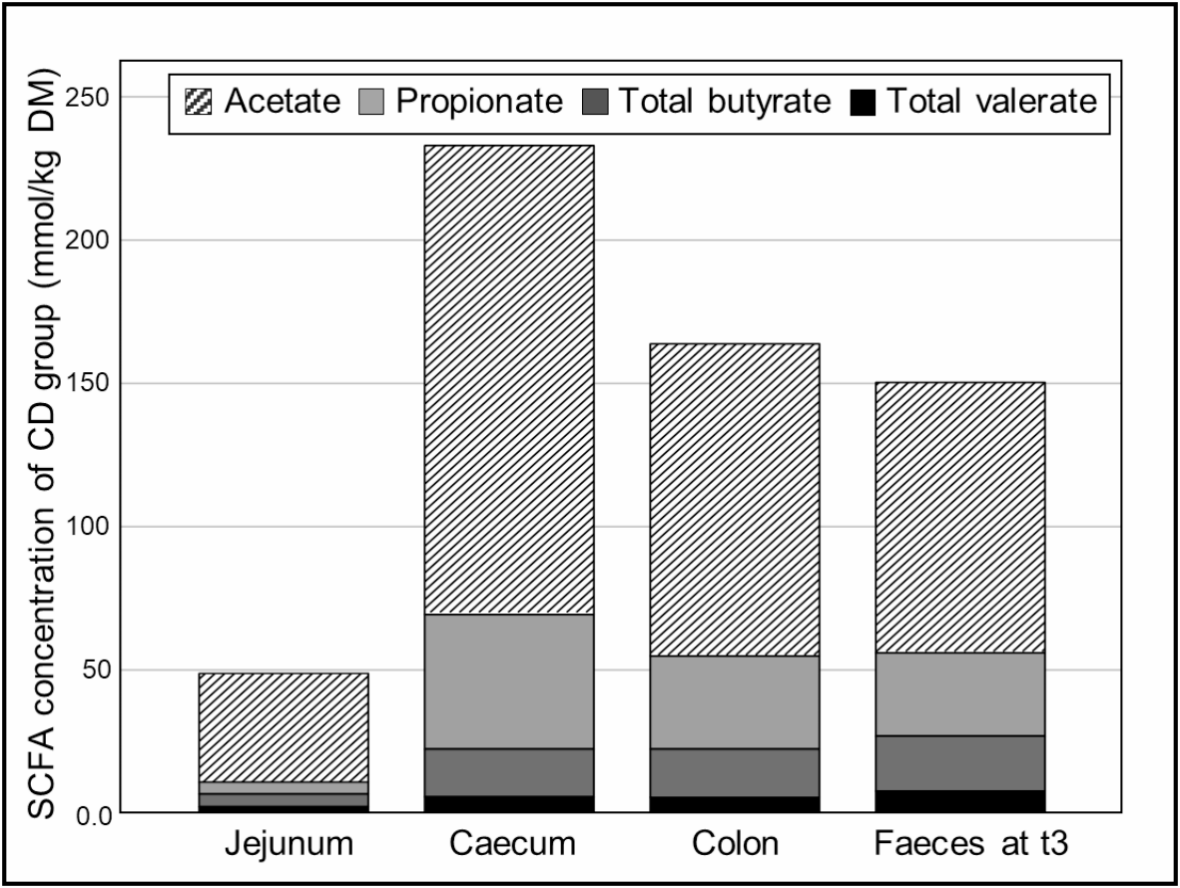
SCFA concentrations (mmol/kg DM) of CD group in chyme and faeces. The data present the SCFA concentrations of CD group in the *jejunum*, *caecum* and *colon* at the time of slaughter and in the faeces at t3. SCFA, short-chain fatty acid; DM, dry matter; CD, group fed the conventional diet (*n* = 9–10).

When the SCFA concentrations were compared between the *jejunum*, *caecum*, ascending *colon* and time point t3 faecal samples, the acetate, propionate, total butyrate and total valerate levels were significantly lower in the *jejunum* than in the *caecum*, *colon* and faeces (*P* ≤ 0.005; Fig. 2). The highest concentrations of acetate and propionate were present in the *caecum*. The SCFA levels in the *colon* and faeces were in a similar range. No significant differences in total butyrate and valerate were found between the *caecum*, *colon* and faeces. Except for faeces, the BL group showed higher concentrations of SCFAs in chyme at all locations than the four feeding groups (AD, ADp, ADi and CD). No significant differences were found between the AD and CD groups or between the AD, ADp and ADi groups in SCFA levels or intestinal location (Table 1 and Fig. 2 in Supplementary File S1). The original data for the pH values and the DM and SCFA content in the faeces and the chyme are provided in a supplementary Table S2.

**Table 1:** SCFA concentrations in Chyme from several intestinal locations and from faeces.

SCFA	Groups	Jejunum	Caecum	Colon	Faeces (t3)
Acetate	BL	76.5^a^ [67.2/83.6]	208 [179/234]	136 [119/139]	n/a
	AD	36.4^#, ab^ [36.4/76.1]	123^▲^ [101/225]	101^▲∎^ [76.2/150]	73.0^#^ [66.1/96.0]
	ADp	46.9^#, ab^ [26.3/65.4]	142^▲^ [120/201]	95.3^■^ [79.5/129]	95.3^■^ [79.5/129]
	ADi	33.0^#, b^ [27.2/40.6]	163^▲^ [138/177]	107^▲■^ [87.9/132]	88.7^■^ [83.7/99.5]
	CD	39.5^#, b^ [25.4/47.8]	178^▲^ [132/206]	111^■^ [81.7/126]	94.5^■^ [86.4/104]
Propionate	BL	4.54 [4.03/4.86]	66.9 [48.6/77.4]	41.8^a^ [34.5/45.7]	n/a
	AD	4.13^#^ [3.73/5.24]	35.8^▲^ [29.5/60.8]	30.2^▲, ab^ [26.0/35.8]	23.4^#▲^ [20.8/28.5]
	ADp	4.87^#^ [3.99/6.10]	46.1^▲^ [35.0/59.3]	25.0^▲■, b^ [21.7/32.0]	24.2^#■^ [21.1/27.9]
	ADi	4.64^#^ [3.75/6.20]	48.2^▲^ [42.3/61.0]	27.5^▲■, ab^ [24.9/35.1]	23.6^#■^ [19.3/29.7]
	CD	3.35^#^ [2.82/5.71]	49.5^▲^ [38.0/59.6]	31.8^▲, ab^ [26.6/38.0]	30.4^▲^ [26.0/32.8]
Total butyrate	BL	11.8^a^ [9.84/14.4]	35.8^a^ [33.8/38.5]	29.2^a^ [27.0/37.4]	n/a
	AD	4.45^#, b^ [4.23/5.64]	12.9^▲, b^ [9.65/21.5]	12.6^▲, b^ [11.3/15.4]	14.2^▲^ [12.2/16.3]
	ADp	4.86^#, ab^ [4.21/5.75]	12.3^▲, b^ [10.8/20.0]	11.3^▲, b^ [10.8/15.0]	14.6^▲^ [11.0/17.2]
	ADi	4.05^#, b^ [3.43/5.03]	14.5^▲, b^ [13.3/15.9]	15.8^▲, ab^ [14.3/18.1]	15.2^▲^ [13.7/18.1]
	CD	4.20^#, b^ [2.62/5.69]	17.1^▲, b^ [11.8/20.7]	16.4^▲, ab^ [12.1/21.2]	18.5^▲^ [16.3/24.3]
Total valerate	BL	2.79 [2.30/3.74]	5.92 [5.61/7.07]	6.82^a^ [5.91/7.54]	n/a
	AD	2.24^#^ [1.96/2.74]	6.13^▲^ [5.29/8.73]	5.67^▲, ab^ [4.44/5.98]	6.84^▲^ [5.79/7.42]
	ADp	3.04^#^ [2.36/3.25]	6.08^▲^ [5.12/7.27]	5.35^#▲, b^ [4.23/5.87]	6.66^▲^ [6.34/7.13]
	ADi	2.67^#^ [2.15/3.32]	7.87^▲^ [6.34/10.6]	6.33^▲, ab^ [5.16/7.83]	6.95^▲^ [4.98/8.87]
	CD	1.81^#^ [1.39/2.99]	5.34^▲^ [4.95/6.76]	5.82^▲, ab^ [4.52/6.06]	7.97^▲^ [7.34/8.34]

SCFA concentrations (mmol/kg DM) of CD group in chyme and faeces. The data present the SCFA concentrations of CD group in the *jejunum*, *caecum* and *colon* at the time of slaughter and in the faeces at t3. SCFA, short-chain fatty acid; DM, dry matter; CD, group fed the conventional diet (*n* = 9–10).

The SCFA concentrations in the *jejunum*,* caecum* and *colon* at the time of slaughter and in faeces at t3 in all groups (BL, AD, ADp, ADi and CD) are presented as medians and [25th/75th] percentiles in mmol/kg DM. ^▲#■^Different symbols indicate significant differences within a row (intestinal section differences in one group). ^ab^Different lowercase letters indicate significant effects within a column (group differences in one intestinal section). Significant differences are identified by P values ≤ 0.05 via repeated-measures ANOVA with the Games–Howell test (acetate) and the Kruskal–Wallis test with Bonferroni correction (propionate, total butyrate and total valerate). SCFA, short-chain fatty acid; DM, dry matter; BL, baseline group (*n* = 6–8); AD, group fed the atherogenic diet (*n* = 9–10); ADp, group fed the atherogenic diet + pectin (*n* = 9–10); ADi, group fed the atherogenic diet + inulin (*n* = 9–10); CD, group fed the conventional diet (*n* = 9–10); n/a, not available.

### Liver fat content did not increase by atherogenic diet

The crude lipid (CL) content of liver tissue (Table 2) was within the physiological range for pig liver^46^ and was similar between the BL group and the feeding groups (AD, ADp, ADi and CD). Between AD and CD groups no significant difference was found for CL. Focusing on the AD, ADp and ADi groups, a significant difference was determined only in *the lobus hepatis dexter lateralis* between ADp and ADi groups with lower values in ADi group (*P* = 0.002). There was also a significant difference in *the lobus hepatis dexter lateralis* between the BL group and the AD, ADp, and CD groups (*P* = 0.002). The original liver fat content data are provided in a supplementary table S2.

**Table 2:** CL content in the liver tissue of the BL group and of each dietary group (% in DM).

Liver tissue	BL	AD	ADp	ADi	CD
Left liver lobe	9.62 ± 1.12	9.67 ± 0.81	10.1 ± 0.99	10.5 ± 1.13	10.3 ± 1.70
Right liver lobe	8.89 ± 1.17 ^a^	10.6 ± 0.60^bc^	10.9 ± 1.88^c^	9.60 ± 0.80^ab^	11.1 ± 1.15^c^

The data are presented as the means ± SD in percentages of DM. ^abc^Different lowercase letters indicate significant differences between the groups, identified by P values ≤ 0.05 via ANOVA with Fisher’s LSD test. Left liver lobe, *lobus hepatis sinister lateralis*; right liver lobe, *lobus hepatis dexter lateralis*; CL, crude lipid; DM, dry matter; BL, baseline group (*n* = 8); AD, group fed the atherogenic diet (*n* = 10); ADp, group fed the atherogenic diet + pectin (*n* = 10); ADi, group fed the atherogenic diet + inulin (*n* = 10); CD, group fed the conventional diet (*n* = 10).

### Inflammatory markers in liver differed markedly between lobes

**Fig. 3:**
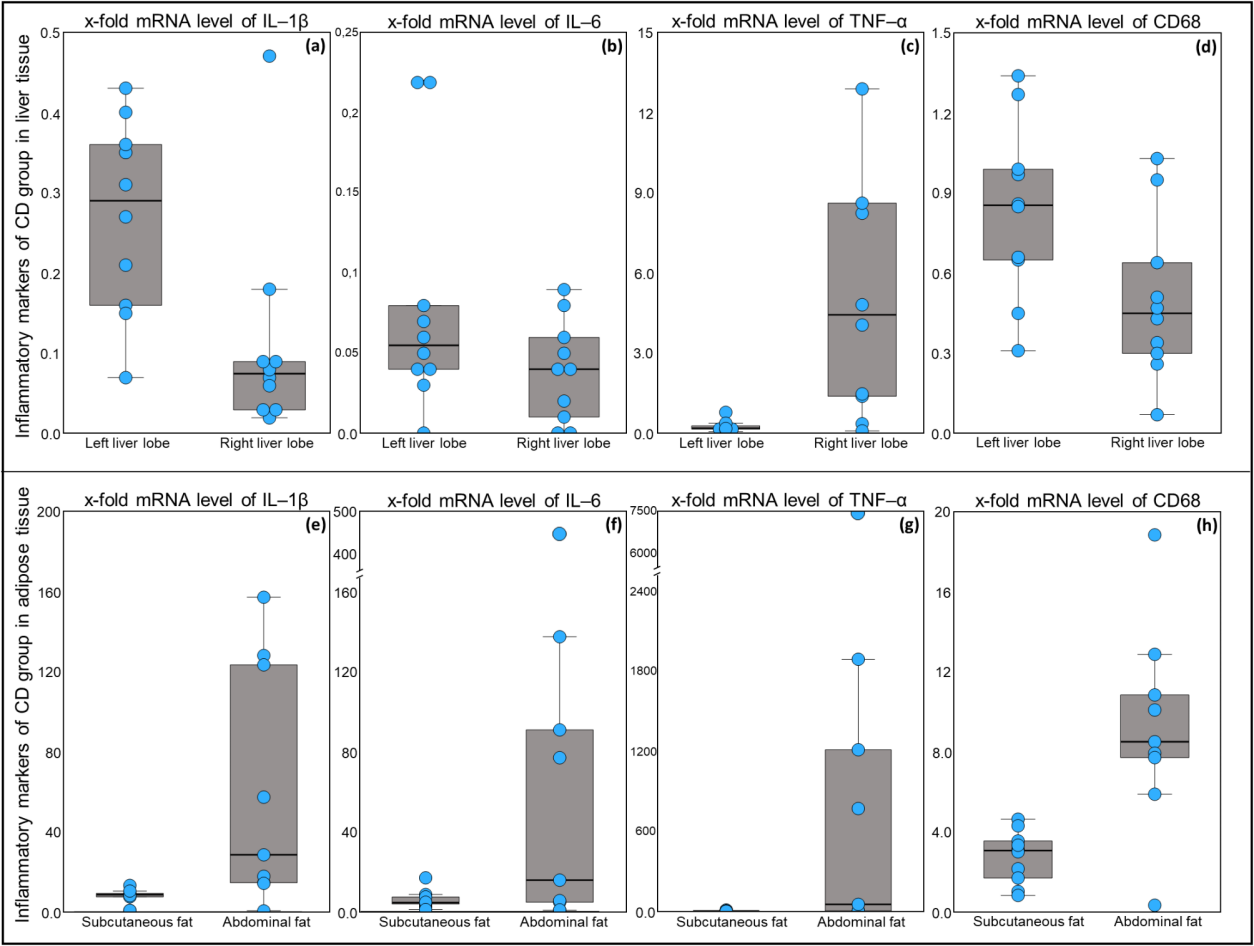
mRNA levels of *IL-1β*, *IL-6*, *TNF-α* and *CD68* in the liver (**a**–**d**) and adipose tissue (**e**–**h**) of CD group. The data are presented as box plots with a single dot per animal. Left liver lobe, *lobus hepatis sinister lateralis*; Right liver lobe, *lobus hepatis dexter lateralis*; IL-1β, interleukin-1β; IL-6, interleukin-6; TNF-α, tumour necrosis factor α; CD68, cluster of differentiation 68; CD, group fed the conventional diet (*n* = 10).

**Fig. 4:**
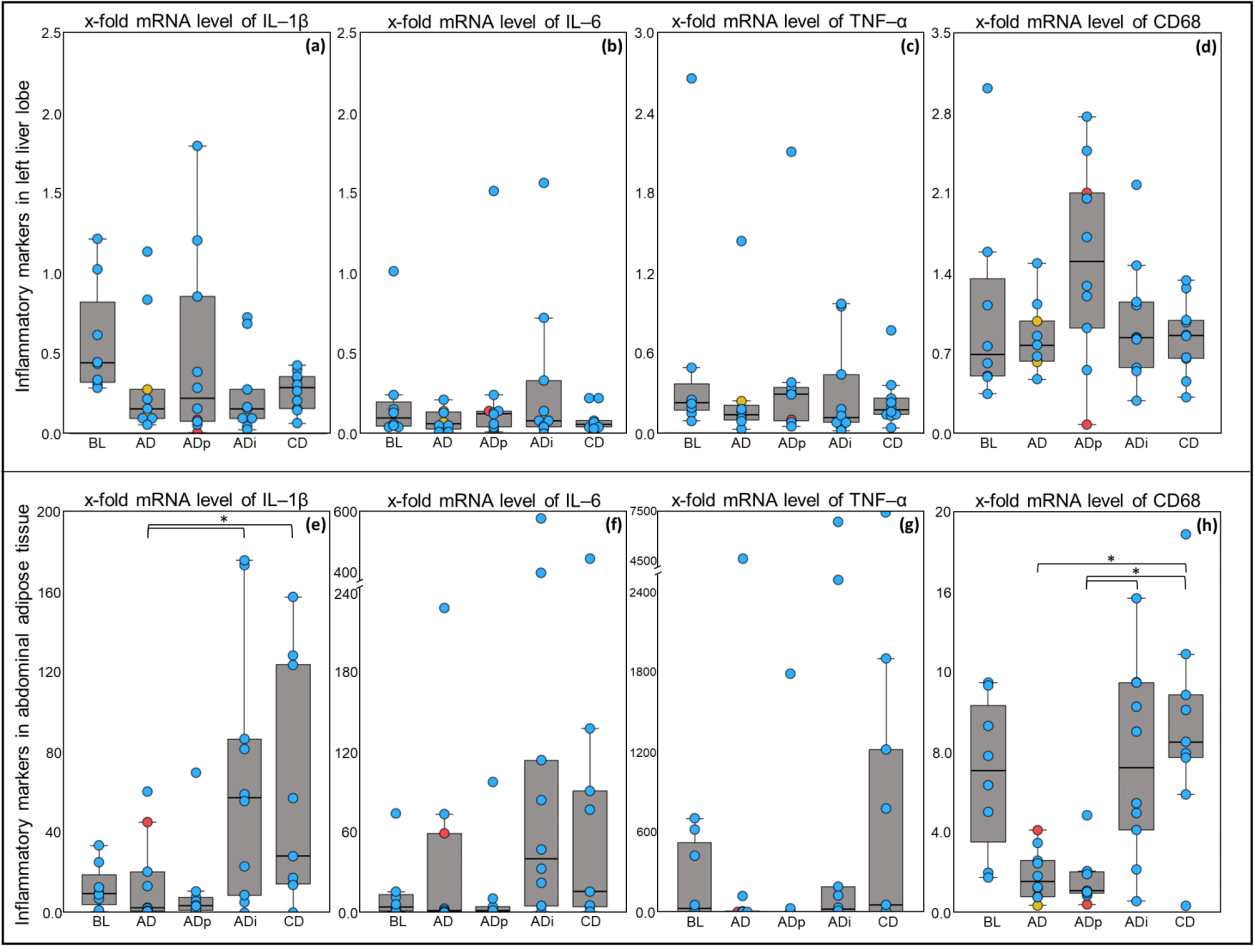
mRNA levels of *IL-1β*, *IL-6*, *TNF-α* and *CD68* in left liver lobe (**a**–**d**) and abdominal adipose tissue (**e**–**h**) of all groups. The data are presented as box plots with a single dot per animal. Animals treated with drugs are marked with red dots for nonsteroidal anti-inflammatory drugs and yellow dots for antibiotics. *Asterisks indicate significant differences between the groups. Significant differences were identified by *P* values ≤ 0.05 via ANOVA with Fisher’s LSD for *IL-1β*, *IL-6* (left liver lobe) and *TNF-α* (left liver lobe) and the Kruskal–Wallis test with Bonferroni correction for *IL-6* (abdominal fat), *TNF-α* (abdominal fat), and *CD68*. IL-1β, interleukin-1β; IL-6, interleukin-6; TNF-α, tumour necrosis factor α; CD68, cluster of differentiation 68; BL, baseline group (*n* = 7–8); AD, group fed the atherogenic diet (*n* = 8–10); ADp, group fed the atherogenic diet + pectin (*n* = 9–10); ADi, group fed the atherogenic diet + inulin (*n* = 9–10); CD, group fed the conventional diet (*n* = 9–10).

In the liver, the relative mRNA levels differed between the *lobus hepatis sinister lateralis* and the *lobus hepatis dexter lateralis* (Fig. 3a–d). The relative mRNA levels of *IL-1β* and *IL-6* in the liver were on average < 1 and did not significantly differ between the groups. For *TNF-α*, the relative mRNA levels in the right liver lobe, but not in the left lobe, differed significantly between the groups (*P* = 0.007; Fig. 3 in Supplementary File S1). Relative mRNA levels of *TNF-α* in the right liver lobe were significant lower in AD, than in CD group (*P* = 0.001). Between the groups, in which the diet changed to an atherogenic diet after t0, significantly lower *TNF-α* levels were found in the AD group compared to the ADi group (*P* = 0.01). The relative *TNF-α* mRNA levels in the left liver lobe were on average < 1 and did not differ between the groups. The mean *cluster of differentiation 68 (CD68)* mRNA level in the left liver lobe exceeded on average 1 in the BL group and the ADp group, whereas all the levels in the right liver lobe were on average < 1. No significant effects were found for *CD68* mRNA levels in the liver (Fig. 4a–d).

### Abdominal adipose tissue showed higher inflammatory marker expression and influences by diet

The relative mRNA levels of inflammatory markers in the adipose tissue differed between subcutaneous and abdominal adipose tissue, with considerably higher levels in the abdominal adipose tissue, excluding *CD68* in the AD and ADp groups (Fig. 3e–h). Inflammatory marker expression was greater in the abdominal adipose tissue than in the subcutaneous adipose tissue, as follows: *IL-1β*: 1.36 to 8.09-fold, *IL-6*: 2.20 to 15.7-fold and *TNF-α*: 10.8 to 433-fold. The levels of *CD68* in the BL, ADi and CD groups were 2.45 to 3.33 times higher in the abdominal adipose tissue than they were in the subcutaneous adipose tissue, whereas in the AD and ADp groups, the *CD68* levels were half as high in the abdominal adipose tissue as they were in the subcutaneous adipose tissue.

In the abdominal adipose tissue the *IL-1β* mRNA levels were significantly lower in AD than in CD group (*P* = 0.03; Fig. 4e). In the groups fed an atherogenic diet, the *IL-1β* mRNA levels were significant higher in ADi group than in AD and ADp groups (*P* = 0.02). Similar results were found for the mRNA levels of *CD68* in the abdominal adipose tissue, with significantly lower *CD68* mRNA levels in the AD group than in the CD group (*P* = 0.02; Fig. 4h). Comparing the groups fed an atherogenic diet, IL-1β mRNA levels were significantly higher in the ADi group than in the ADp group (*P* = 0.03). The mRNA levels of *IL-6* in the abdominal adipose tissue showed no significant differences between AD and CD groups, but tended to be higher in the ADi group compared to AD and ADp groups (*P* = 0.087; Fig. 4f). The *TNF-α* mRNA levels in abdominal adipose tissue were numerically higher in the CD group than in the AD group (*P* = 0.087), but no significant differences were found between the groups fed an atherogenic diet (Fig. 4g).

In the subcutaneous adipose tissue, no significant differences were observed between AD and CD group or between the groups fed the atherogenic diet. A significantly lower IL-6 mRNA level was found in the BL group compared to ADi (*P* = 0.02). Overall, a large animal-individual variation was observed for inflammatory mRNA expression in the different tissues, namely in the adipose tissue (Fig. 4 in Supplementary File S1). Original data on inflammatory markers in liver and adipose tissue are provided in a supplementary table S2.

### Number of macrophages in adipose tissue were not affected by diet

**Fig. 5:**
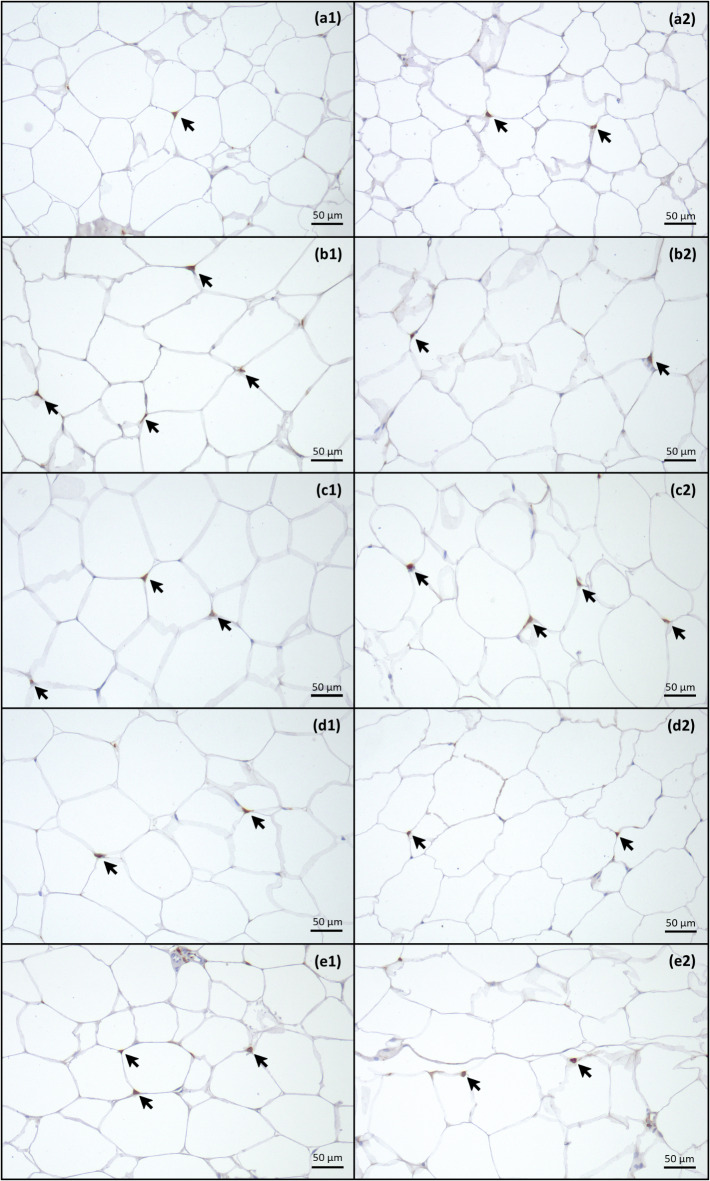
Iba-1-positive macrophages in adipose tissue. Presented are two images per group [BL group (**a**); AD group (**b**); ADp group (**c**); Adi group (**d**); CD group (**e**)] of the subcutaneous (1) and abdominal adipose tissue (2) with 20x magnification. Arrows mark brown-stained macrophages. BL, baseline group; AD, group fed the atherogenic diet; ADp, group fed the atherogenic diet + pectin; ADi, group fed the atherogenic diet + inulin; CD, group fed the conventional diet.

The number of Iba-1-positive macrophages per mm^2^ (Fig. 5) in the abdominal and subcutaneous adipose tissue did not significantly differ between the groups (*P* = 0.3). There was only a trend in the subcutaneous adipose tissue to a greater number of macrophages in the AD and ADp groups than in the BL group (*P* = 0.09; Table 3). The original data concerning the number of macrophages in the adipose tissue are provided in a supplementary table S2.

**Table 3:** Number of macrophages in adipose tissue (mean number/mm^2^).

Adipose tissue	BL	AD	ADp	ADi	CD
Subcutaneous fat	4.13 ± 2.36	9.64 ± 5.13	7.54 ± 3.61	5.84 ± 3.29	5.35 ± 2.71
Abdominal fat	6.21 ± 2.31	7.40 ± 4.00	7.31 ± 2.96	5.78 ± 1.93	5.24 ± 4.24

The data are presented as the means ± SDs of the number of macrophages/mm^2^. Significant differences were identified by P values ≤ 0.05 via Kruskal–Wallis test with Bonferroni correction. BL, baseline group (*n* = 8); AD, group fed the atherogenic diet (*n* = 10); ADp, group fed the atherogenic diet + pectin (*n* = 10); ADi, group fed the atherogenic diet + inulin (*n* = 10); CD, group fed the conventional diet (*n* = 10).

## Discussion

In the present study, selected inflammatory markers in the liver as well as in the abdominal and subcutaneous adipose tissues were compared between different feeding groups. We used a Saddleback pig model under well-defined feeding and housing conditions as a model for human obesity and overnutrition. The atherogenic diet applied in this study was comparable to a typical Western-style diet in humans. The diet was supplemented with 5% (w/w) pectin or 5% (w/w) inulin in two feeding groups. We hypothesized that supplementing an atherogenic diet with fermentable carbohydrates would have lowering effects on the inflammatory response in obese pigs by modulating SCFA production.

While the faecal pH values of all the groups were within a similar range before the start of the feeding period, the faecal pH values of the groups fed an atherogenic diet supplemented with pectin and inulin (ADp, ADi) were significantly lower than those of the conventionally fed group (CD) one month after the different diets were administered (*P* = 0.01). One likely explanation is related to the proportion of carbohydrates (pectin, inulin) in the diet that could be fermented into SCFAs. In other studies conducted in pigs, the addition of 5% (w/w) pectin or 4% (w/w) inulin increased the concentration of caecal SCFAs^47,48^. However, over the entire feeding period, the faecal pH increased significantly in all the feeding groups after they were fed the different diets (*P* < 0.04). The increase in faecal pH was associated with a reduction in total faecal SCFA levels in all the feeding groups over the feeding period. These results contrast with those of other studies in pigs in which an increase in SCFA levels in the intestine caused by supplementation with 4% (w/w) inulin or 5% (w/w) pectin was reported^47,48^. However, Tian et al.. (2017) found no effects on faecal SCFA concentrations or proportions with 3% (w/w) pectin supplementation in pigs^49^. Particularly in the groups that had a feed change to an atherogenic diet (AD, ADp, ADi) after t0, significant reductions in the levels of faecal SCFAs were observed, excluding acetate and valerate levels in the ADi group. At t1, the faecal acetate levels were significantly lower in the ADp group than in the CD group (*P* < 0.01). As acetate is the most abundant SCFA, this contradicts the theory that the pectin supplementation at the same time point could be the cause of the significant pH difference (*P* = 0.01) between the ADp (pH 6.60 [6.51/6.73]) and CD (pH 6.93 [6.80/7.13]) groups. The more pronounced lowering of faecal SCFA levels in the atherogenic-fed groups might have occurred because the high fat content in the atherogenic diet may have negatively affected the fermentation processes of the intestinal microorganisms. The high fat content in the atherogenic diet and the possible negative impact on the intestinal microflora were reflected by the markedly increased fat content in the faeces of the pigs after the start of the feeding period, as described by Wahl et al.. (2022)^45^. Before the beginning of the feeding period (t0), the fat content in the faeces of all the feeding groups was < 7.5% in the DM. After the administration of the atherogenic diets for one month (t1), the faecal fat content increased to 20–25% in the DM (AD, ADp, ADi), whereas the faecal fat content in the CD group decreased to < 5% in the DM. In accordance with our study, Heinritz et al.. (2016) reported significantly lower faecal SCFA concentrations in pigs fed a high-fat diet than in those in another group fed a low-fat, high-fibre diet^16^. In the comparison of the total SCFA concentration between different intestinal sections (*jejunum*, *caecum* and ascending *colon*) and faeces at the end of the study (t3), the lowest SCFA concentration was found in the *jejunum*, and the highest SCFA concentration was found in the *caecum*. The highest concentrations of acetate and propionate were detected in the *caecum*, whereas no significant differences in total butyrate and valerate were found among the *caecum*, *colon* and faeces. This result corresponds to the conditions in the human intestine, where the main fermentation of carbohydrates to SCFAs takes place in the proximal *colon*^25^. The SCFA levels in the *colon* and faeces are comparable, therefore, faecal samples, as a noninvasive tool, can be used to reflect SCFA production in the distal *colon*^23^. However, it should be considered that only 5–10% of SCFAs are eliminated in faeces^25^. Interestingly, the BL group had higher concentrations of total and individual SCFAs in all the intestinal sections than the other groups, which may have been caused by the greater rate of SCFA absorption in the intestine due to maturation of the digestive tract in the older pigs at the end of the study. A study in humans showed that ageing has a major influence on the faecal SCFA concentration, with higher levels found in younger subjects^50^.

With respect to the liver fat content, the fat content of pigs in all the groups was within the physiological range, and no hepatic steatosis developed^46^. There were no significant differences between the groups in terms of liver fat content in the left liver lobe. However, in the right liver lobe, there were lower values in the BL and ADi groups (*P* < 0.01). Differences between the sides of the liver were also found for the expression of inflammatory markers. While higher relative mRNA expression levels of *IL-1β*, *IL-6* and *CD68* were detected in the left liver lobe in all groups without significant group differences, the relative mRNA levels of *TNF-α* in the right liver lobe were greater. The *TNF-α* expression levels differed significantly between the AD group and the CD group with lower expression levels in AD group (*P* = 0.001). The AD group also showed lower *TNF-α* expression levels than ADi group in the right liver lobe (*P* = 0.01). The values for *IL-1β* and *IL-6* in the left liver lobe were on average less than 1, and for *CD68*, the mean value of 1 was only slightly exceeded in the BL and ADp groups. In the case of *TNF-α* in the right liver lobe, there were relevant mRNA expressions with mean values > 4 in the BL, ADp, ADi and CD groups. The heterogeneous distribution of regional portal blood flow in the liver could have been a critical factor, as observed in a study in pigs by Thein et al.. (2003)^51^. This study revealed significantly greater values for regional portal blood flow in the area near the diaphragm and significantly lower values in the left liver lobe than in the central and right liver lobes^51^. In accordance with our study, Merriman et al.. (2006) reported only a moderate similarity between the inflammatory responses in biopsy samples from the right and left liver lobes of patients with nonalcoholic fatty liver disease^52^. Therefore, variations in the levels of the same parameter between different liver lobes must be accounted for in the interpretation.

The relative mRNA levels of inflammatory markers in the abdominal adipose tissue were up to 400-fold higher than those in the subcutaneous adipose tissue. This result is consistent with other studies in humans and pigs, which also reported greater expression of inflammatory markers in the abdominal adipose tissue than in the subcutaneous adipose tissue and emphasized the similarity between humans and pigs, suggesting that pigs are a suitable obesity model for humans^3,5,6,8,9^. In addition, significant differences in the levels of inflammatory markers in the subcutaneous adipose tissue were detected only between BL and ADi for IL-6 with lower expression levels in BL group (*P* = 0.02). These results are in line with the findings of other researchers, since abdominal adipose tissue, in particular, is attributed a high inflammatory potential, whereas subcutaneous adipose tissue seems to have lower inflammatory activity and is even associated with protective effects^3,5,6,53^. For example, subcutaneous adipose tissue has been identified as the main source of leptin^54^, and subcutaneous adipose tissue, particularly that in the gluteofemoral region, is attributed to have a protective potential against lipotoxicity and the formation of ectopic fat deposits^53^. In the abdominal adipose tissue, in contrast to the subcutaneous adipose tissue, there were significant differences in the expression levels of inflammatory markers between the feeding groups.

For example, the AD group showed significantly lower *IL-1β* (*P* = 0.03) and *CD68* (*P* = 0.02) mRNA levels in the abdominal adipose tissue than the CD group. When comparing the groups fed an atherogenic diet, the expression levels of *IL-1β* and *CD68* were significantly lower in the ADp group than in the ADi group (*P* ≤ 0.03). However, the same findings were found for the AD group, which received no supplementation of fermentable carbohydrates. With regard to *IL-1β*, the expression levels of the AD group were also significantly lower than those of the ADi group (*P* = 0.02).

The present findings do not support our hypothesis that pectin supplementation of an atherogenic diet posed some benefits in terms of the inflammatory response of abdominal adipose tissue in obese pigs. Therefore, our study did not confirm the results of other studies, which demonstrated an anti-inflammatory potential of pectin in humans and other species, such as pigs^33–37,48^. For example, a study in obese rats revealed a decrease in the mRNA levels of *IL-6* and *TNF-α* in the *ileum* and serum by supplementing 5% pectin to a high-fat diet for six weeks^36^. In two other studies in pigs, the LPS-induced inflammatory response in the *ileum* (mRNA level of *IL-1β*, *TNF-α*) and *caecum* (mRNA level of *IL-1β*, *IL-6*, *TNF-α*) was significantly reduced by supplementation with 5% pectin for three weeks^37,48^. Interestingly, even the feeding group that received the atherogenic diet without supplementation (AD) showed similarly low expression levels of inflammatory markers as the ADp group did. In the AD and ADp groups, lameness occurred more frequently during the feeding period (AD: 30% lameness in animals, ADp: 20% lameness in animals) than in the other groups (ADi: 0% lameness in animals, CD: 0% lameness in animals). An likely explanation could be the treatment of the animals with anti-inflammatory drugs which may reduce the mRNA levels of inflammatory markers in these groups. However, comparison of data between the treated and untreated animals showed no drug treatment effects on the expression of inflammatory markers. Therefore, the time lag of at least two weeks between the last day of medication and sampling can be considered sufficient to avoid drug interference on the outcome of inflammatory response. In addition, strong animal-individual variation was observed in inflammatory response of adipose tissue. It is possible that a longer experimental period in which the animals had been in a chronic obese state for a longer time period would have triggered low-grade tissue inflammation in more animals. However, the metabolically healthy obese phenotype has also been recognised in humans for decades, which develops less inflammation in the adipose tissue, particularly in the abdominal adipose tissue^55^.

In contrast to other studies that used lower amounts of inulin and shorter supplementation periods, inulin supplementation had no beneficial effects on the inflammatory response in our study^29–32^. For example, supplementation with 12 g of inulin over four weeks in humans was able to reduce TNF-α levels in the peripheral blood^31^. In a study conducted in pigs, the expression of *TNF-α* in the *colon* was reduced by supplementation with 4% (w/w) inulin for 5–7 weeks^32^. However, the abovementioned studies did not use a high-fat diet, and the inflammatory markers were examined in other tissues, such as blood or chyme.

The macrophage levels in the adipose tissue showed no significant group differences in either the subcutaneous or the abdominal adipose tissue, which may indicate that the absolute invasion of macrophages into adipose tissue is not a determinant of the relative expression of inflammatory mediators. In the present study, the inflammatory responses of the abdominal adipose tissue may have originated less from migrated proinflammatory macrophages than from the adipocytes themselves. As described in the literature, adipocytes produce and secrete more than 600 different adipokines, including many proinflammatory cytokines such as IL-1β, IL-6 and TNF-α^5^. In contrast to our results, other studies in obese humans and pigs revealed an increased number of macrophages in abdominal adipose tissue compared with subcutaneous adipose tissue^10,56^. In a study conducted by Cirera et al.. (2020) in Göttingen minipigs, no increase in macrophage infiltration into the abdominal or subcutaneous adipose tissue was found with the administration of an atherogenic diet over 13 months, a finding that is comparable to our results^57^. However, no upregulation of the expression profiles of proinflammatory cytokines could be detected except for IL-6 in the subcutaneous adipose tissue in Göttingen minipigs^57^.

Although differences were observed between the feeding groups in terms of inflammatory responses in abdominal adipose tissue, the diet with fermentable carbohydrate supplementation did not resolve the problem of low-grade tissue inflammation associated with chronic positive energy balance and resulting obesity. Lifestyle modifications, including a well-balanced diet, remain essential to manage obesity.

## Materials and methods

### Animals and feeding

In this study, 48 healthy Saddleback pigs owned by the Institute of Animal Nutrition, Nutrition Diseases and Dietetics, Leipzig University, were included. All pigs were healthy and in a good body condition at the beginning of the study. The animal cohort at the age of five months included 21 female and 27 castrated male pigs from five litters with the same sire. The pigs were randomly divided into four feeding groups of ten animals each (atherogenic diet = AD, atherogenic diet + 5% (w/w) pectin = ADp, atherogenic diet + 5% (w/w) inulin = ADi, and conventional diet = CD) and one baseline group of eight animals that were slaughtered at the beginning of the study (BL). The pigs were housed in the same barn, separated into one pen per group according to the allotted treatment and bedded on wood shavings. Before the beginning of the study, the animals were adapted to the general experimental environment and fed the same conventional diet for at least three weeks: 43.5% wheat, 38.0% oat, 14.0% soy bean meal, 4.50% vitamins, minerals and fibre supplement and metabolizable energy (ME) of 14.1 MJ/kg DM. After an adaptation period, the BL pigs were slaughtered to obtain baseline values of liver, adipose tissue and chyme samples. The remaining groups were fed treatment diets ad libitum for 15 weeks. The atherogenic diet was composed of 10.0% wheat, 18.0% oat, 17.5% soybean meal, 4.50% vitamins, minerals and fibre supplements, 38.0% crisps, 10.0% palm fat, 2.00% sugar and ME of 17.8 MJ/kg DM. Water was provided ad libitum via an automatic watering system. Further information on housing and feeding conditions is provided in Wahl et al.. (2022)^45^. At the end of the feeding period, the pigs were slaughtered for sample collection. This project was approved by the Ethics Committee for Animal Rights Protection of the Leipzig District Government (no. TVV 04/20) in accordance with German legislation for animal rights and welfare and the ARRIVE guidelines. Supporting information on the project and a detailed composition of the treatment diets are provided in a supplementary file S1.

### Health monitoring and morphometric measurements

Health checks of each pig were performed at two-day intervals via clinical examination, including evaluations of general behaviour, feed and water intake, faecal quality, breathing rate, and body temperature. In addition, blood tests (blood count and chemistry) were performed before and after the feeding period. During the feeding period, the daily feed intake of each group was recorded. Body weight was measured weekly using a portable electronic scaling system (Minipond 21; Baumann Waagen und Maschinenbau, Thiersheim, Germany). Body condition was evaluated weekly via a score ranging from 0 to 5^58^. Back fat thickness was obtained monthly by transcutaneous ultrasound measurements (Portable Ultrasonic Diagnostic System A6V, SonoScape, Shenzhen, China) at six measurement points according to the ABC-6 method^59^. The results of these parameters are presented in Wahl et al.^45^ and a supplementary file S1.

### Blood sampling

Samples for blood chemistry were collected at the beginning of the study (t0; October 8–13, 2020). Follow-up blood samples were collected after one (t1), two (t2), and three months (t3) of feeding the experimental diets. The blood sampling procedure is described in Wahl et al.. (2022)^45^ and a supplementary file S1.

### Faecal sampling

Rectal faecal samples from each animal were collected at the same time points (t0–t3) as the blood samples. At each time point, a 13 g sample was frozen at -80 °C for later analysis of SCFAs. In addition, the pH was determined using a pH meter (PHM 93 Reference pH meter, Radiometer Copenhagen, Bagsvaerd, Denmark) immediately after sampling. Afterwards, the DM of the faeces was determined after oven drying (103 °C).

### Slaughtering of the pigs and sampling of liver, adipose tissue and Chyme

The pigs were stunned using electrical stunning equipment (TGB 200; Hubert Haas, Neuler, Germany). Blood was drawn immediately after electrical stunning by severing the brachiocephalic trunk and jugular vein. The pigs were slaughtered in accordance with European and German law [Council Regulation (EC) No 1099/2009 of 24 September 2009, Tierschutz-Schlachtverordnung, § 4 Tierschutzgesetz]. After slaughter, half of the *lobus hepatis sinister lateralis* and *lobus hepatis dexter lateralis* and the gastrointestinal tract in toto were removed for subsequent sampling. Immediately, 5 g of each liver lobe was obtained aseptically, shock-frozen in liquid nitrogen and stored at -80 °C until analysis of inflammatory markers. Adipose tissue was collected from two locations. Subcutaneous adipose tissue was harvested from the back fat located above the 7th cervical vertebra. Abdominal adipose tissue was harvested from the area of the *mesocolon ascendens*. As described for the liver, 5 g of each adipose tissue sample was immediately shock-frozen. Two other pieces of 1 cm^3^ were fixed in 4% neutral-buffered formaldehyde (OMEGA Pharma Deutschland, Herrenberg, Germany) for at least two days to prepare polyethylene glycol sections as well as for immunohistochemical examination. After the adipose tissue was removed, chyme was taken from the middle of the *jejunum*, *caecum*, and *colon ascendens*. Samples of 13 g in duplicate were collected in tubes from each of the three intestinal locations. The chyme pH was determined immediately with a pH meter (PHM 93 Reference pH meter, Radiometer Copenhagen), and the DM content of the chyme was determined after oven drying (103 °C). Chyme samples were frozen at -80 °C for further analysis of SCFAs.

### Analyses

### Serum parameters of liver and lipid metabolism

TG, BA, LIPC, CHOL, ALP, AST, GGT, LDH and AMYL levels were analysed via an automated chemistry analyser (Roche Cobas C311, Roche Diagnostic, Mannheim, Germany). Blood counts were evaluated in all pigs before and after the 15-week feeding period using an ADVIA 120 (Siemens Healtheneers, Dreieich, Germany).

### SCFAs in Chyme and faeces

For the analysis of SCFA acetate, propionate, butyrate, isobutyrate, valerate, isovalerate, caproate and isocaproate in faeces and chyme, 2.5 g of each sample was weighed and diluted with 1 mL of phosphate-buffered saline (PBS). After ultrasonication for approximately 10 min, the faeces-PBS mixture was centrifuged for 2 h at 50,000 U/min (Rotor type 50.4 ti, Beckman Coulter, Brea, CA, USA). Afterwards, the solid phase was separated, and 500 µL of the supernatant was stored at -80 °C. For the analysis of SCFAs, samples were thawed and mixed before 10 µL of i-caproic acid (internal standard) was added to 500 µL of the supernatant. The samples were mixed and measured via gas chromatography. For gas chromatographic measurements, 1 µL of the solution was injected into a Shimadzu model GC 17 A (Shimadzu, Kyoto, Japan). SCFA analysis was adapted to the procedure reported by Roessler et al.^60^ and Klinder et al.^61^.

### Fat content of the liver tissue

The amount of CL was determined in liver samples from the four feeding groups (AD, ADp, ADi, and CD) and the BL group. The CL content in the liver tissue was analysed by using the Weende system^62^.

### mRNA levels of inflammatory cytokines in the liver and adipose tissue

The quantity of mRNA transcripts in the liver and adipose tissue was determined via quantitative real-time PCR. A commercial kit (RNeasy Lipid Tissue Mini Kit, QIAGEN, Hilden, Germany) was used to isolate total RNA according to the manufacturer´s protocol. The RNA quantity and purity were measured via spectrophotometry (NanoDrop One, Thermo Fisher Scientific, Darmstadt, Germany). The quality of the RNA was determined using gel electrophoresis and a fragment analyser (Agilent Technologies, Santa Clara, CA, USA). The RNA samples were transcribed into cDNA by using two mastermixes (mastermix I: random primer, deoxynucleotide triphosphates (5 min at 65 °C in a thermal cycler); mastermix II: 5x First Strand Buffer, dithiothreitol, SuperScript II Reverse Transcriptase; Thermo Fisher Scientific) and a standardized protocol (12 min at 25 °C, 50 min at 42 °C, 15 min at 70 °C, then 4 °C) with a thermal cycler (Biometra TRIO, Göttingen, Germany). cDNA was stored at -20 °C until analysis, and the quality of the cDNA was ensured via gel electrophoresis. The genes of interest included the following markers of inflammation: IL-1β, IL-6, TNF-α and CD68. Gene sequences encoding the genes of interest were obtained from the Ensembl database (https://www.ensembl.org). The 18 S ribosomal RNA gene (18 S), glyceraldehyde 3-phosphate dehydrogenase (GAPDH), peptidylpropyl isomerase A (PPIA) and ß-actin were tested as reference genes according to the literature^63–66^, and 18 S was selected. The primer (biomers.net, Ulm, Germany) design and validation followed standard procedures. The lengths of the primers varied between 18 and 20 base pairs, with a minimum guanine and cytosine content of 50–60%. When possible, more than three terminal t bases and more than four repeating structures were avoided. Table 4 provides an overview of the primer sequences used. For quantification of the transcripts, standard curves were generated on each plate with serial dilutions of pooled cDNA from all the samples. Additionally, a no-template control was included in each plate. Quantitative polymerase chain reaction was performed via 45 cycles of a standard ABI program (QuantStudio 6 Flex Real-Time PCR System, Thermo Fisher Scientific) and Power SYBR Green PCR Master Mix (Thermo Fisher Scientific). The amplification of specific transcripts was confirmed by melting curve profiles at the end of each PCR. The genes of interest were normalized against the selected reference gene 18 S.

**Table 4:** Primer sequences used to analyse the mRNA levels of genes of interest and reference genes.

Genes	Forwards (5´-3´)	Backwards (3´-5´)
IL-1β	GCTCCCTACCATTTGGCACT	GAGTTTCCCAGGAAGACGGG
IL-6	GGTGTTCGTTTTGGGAGCAC	AGGAGCTACCTCTGACCAGG
TNF-α	GTCGCCCACGTTGTAGGTAA	ACTCTGCCATTGGAGCTGTC
CD68	CCCTTGCCACACTCCTTAGT	ACCCCTAACCTGGGAACCTT
18 S	GCCCTCGGTCGAGTTGTC	TTGCAGGGCGGTGACAG
GAPDH	GTACGACCACCCCATCCAAG	CCCCGCGATCTAATGTTCTCT
PPIA	ACAACTGCTTCCTTTCCTGCAT	AATGCGATAGGAGCTCTGGC
β-Actin	GCGCAGCAATATCGTCATCC	GGCTTCCTTTGTCCCCAATCT

### Immunohistochemistry for quantification of macrophages in the adipose tissue

Samples obtained at slaughtering were fixed in 4% neutral-buffered formaldehyde (OMEGA Pharma Deutschland, Herrenberg, Germany) over a period of at least 24 h and then embedded in paraffin wax (Engelbrecht Medizin- und Labortechnik, Edermünde, Germany) via a computer-controlled tissue processor (Donatello, DiaPath, Martinengo, Italy) with automated fixation, dehydration, cleaning and paraffin infiltration according to a standard protocol.

Iba-1 immunohistochemistry for quantification of macrophages in the adipose tissue was performed using the avidin-biotin-peroxidase complex (ABC) method, as described previously by Kessler et al.^67^. Therefore, 3–4 μm thick sections were prepared from the paraffin blocks via a sliding microtome (Leica SM2010 R, Leica Mikrosysteme, Wetzlar, Germany). These sections were mounted on Superfrost Plus slides (Menzel-Gläser, Menzel, Braunschweig, Germany), air dried for 10 min at room temperature, and then transferred to a drying oven at 65 °C for 20 more min. For deparaffinization, the slides were washed two times in xylol (Vogel, Beucha, Germany) for 10 min, two times in isopropanol (VWR International, Radnor, PA, USA) for 3 min, and once in 96% ethanol (VWR International) for 3 min. Endogenous peroxidase was blocked by incubating the slides in methanol (VWR International) with 0.5% H_2_O_2_ for 30 min at room temperature (Carl Roth, Karlsruhe, Germany), followed by transfer into tris-buffered saline (TBS, pH 7.6). For antigen retrieval, citrate buffer (pH 6.0) was preheated to 96 °C, wherein slides were incubated for 25 min, followed by cooling at room temperature for 20 min, flushing in TBS, and insertion into plastic cover plates (Thermo Fisher Scientific Waltham, MA, USA). Blocking of nonspecific binding sites was achieved with 100 µl of 5% (v/v) normal goat serum (NGS; S-1000, Vector Laboratories, Newark, CA, USA) in TBS per slide for 20 min at room temperature. Afterwards, 100 µL of the primary rabbit anti-Iba-1 antibody (NBP2-19019; Novus Biologicals, Wiesbaden, Germany) diluted 1:1000 in TBS was added to each slide and incubated overnight at 4 °C. The negative controls were incubated with 100 µL of nonimmune normal rabbit serum (Jackson ImmunoResearch Laboratories, Cambridgeshire, United Kingdom) diluted 1:1000 in TBS per slide. The following day, all the slides were washed twice with TBS for 5 min, followed by incubation with 100 µL of the secondary biotinylated goat-anti-rabbit antibody (BA-1000, Vector Laboratories, Newark, CA, USA) diluted 1:200 in TBS per slide for 30 min at room temperature. Afterwards, the slides were washed again twice with TBS for 5 min.

Immunoreactivity was visualized by incubating each slide with 100 µL of ABC solution (Vectastain PK-6100 Elite ABC-HRP Kit, Vector Laboratories) for 30 min at room temperature. The slides were subsequently washed twice with TBS, placed in a staining jar, and incubated in buffered 3.3’-diaminobenzidine-tetrahydrochloride substrate (DAB; Sigma Aldrich, Steinheim, Germany) for 10 min. After three washes in TBS for 5 min and in deionized water for 5 min, the slides were counterstained with haemalaun for 30 s. Next, the sections were washed with tap water for 10 min. Finally, the slides were dehydrated with an ascending ethanol series followed by isopropanol and xylene and embedded in Roti-Histokitt (Carl Roth) and glass coverslips.

For morphometric analysis, all sections of subcutaneous and abdominal adipose tissue (*n* = 96) were digitalized via a slide scanner (Axioscan 7, Carl Zeiss, Oberkochen, Germany) with ZEN blue software (Carl Zeiss Microscopy GmbH, Jena, Germany), a 20x objective with a numeric aperture of 0.45 and a 0.1725 × 0.1725 μm pixel size of the scanned slides, and stored on a server-based ZEN data storage (Carl Zeiss Microscopy GmbH), as described previously by Landmann et al.^68^. The number of Iba-1-positive macrophages was visually determined in 10 squares of 1 mm^2^ each per slide by using QuPath^69^, and the mean number of macrophages per mm^2^ was calculated. Only squares without vascular structures were used, and only cells that had clear cell borders and intense brown cytoplasmic immunoreactivity were counted as macrophages. All the sections were analysed by a blinded investigator.

### Statistical analysis

Data analysis was performed with commercial statistical software (SPSS Statistics version 27.0, IBM, Armonk, NY, USA and STATISTICA version 14.0, TIBCO, Palo Alto, CA, USA). All the data were z-standardized and checked for a normal distribution via the Shapiro–Wilk test. If the datasets were not normally distributed, the data were logarithmically transformed and retested for a normal distribution. Variance homogeneity was determined using the Levene test. The datasets of propionate in faeces, liver fat content, IL-1β, IL-6, TNF-α, CD68 in the liver (right liver lobe) and IL-1β (abdominal fat) in adipose tissue were normally distributed and homogeneous. Repeated measures ANOVAs were used for these parameters, and as a post hoc test, Fisher’s LSD test was applied. The acetate in chyme and faecal pH data were normally distributed but not homogeneous. Therefore, the Games–Howell test was used as a post hoc test. As the datasets were not normally distributed, the Kruskal‒Wallis test with Bonferroni correction was performed for the following: acetate in faeces, propionate in chyme, total butyrate (= butyrate + isobutyrate), total valerate (= valerate + isovalerate), DM content in faeces, CD68 in liver (left liver lobe), IL‒1β in subcutaneous fat, IL‒6, TNF‒α and CD68 in adipose tissue and the number of macrophages in the adipose tissue. Differences were considered significant at P values of < 0.05. The data are shown as medians and [25th/75th] percentiles for acetate, propionate, total butyrate and total valerate or as mean values ± standard deviations (mean ± SD) for liver fat content and the number of macrophages in the adipose tissue.

## Electronic supplementary material

Below is the link to the electronic supplementary material.

Supplementary Material 1

## Data Availability

The data generated or analysed during study are included in the manuscript. The original data of pH, DM and SCFAs in the faeces and chyme, liver fat content, inflammatory markers in the liver and adipose tissue and the number of macrophages in the adipose tissue are provided as supplementary information.
