# Supplementary material for: Type of diet has no major influence on inflammatory response in a Saddleback pig model
Source: Sci Rep. 2025 Mar 11;15:8381. doi: 10.1038/s41598-025-92420-y (PMC11897318; doi:10.1038/s41598-025-92420-y)
Supplement: Supplementary file 1 — Supplementary Material 1 [file 41598_2025_92420_MOESM1_ESM.pdf]

Type of diet has no major influence on inflammatory response in a Saddleback pig model

Lisa Wahl[1,2], Susanne Rau[3], Christine A. Dawczynski[2,4], Stefan Lorkowski[2,4,5], Reiner Ulrich[3], Matthias Blüher[6], Ingrid Vervuert[1,2]*

[1]Institute of Animal Nutrition, Nutrition Diseases and Dietetics, Leipzig University, Leipzig, Germany
[2]Competence Cluster of Nutrition and Cardiovascular Health (nutriCARD), Halle-Jena-Leipzig, Germany
[3]Institute of Veterinary Pathology, Leipzig University, Leipzig, Germany
[4]Junior Research Group Nutritional Concepts, Institute of Nutritional Sciences, Friedrich Schiller University Jena, Germany
[5]Institute of Nutritional Sciences, Friedrich Schiller University Jena, Germany
[6]Helmholtz Institute for Metabolic, Obesity and Vascular Research (HI-MAG) of the Helmholtz Zentrum München at Leipzig University and University Hospital Leipzig, Leipzig, Germany

S1: ARRIVE guidelines 2.0 Checklist with detailed composition of the treatment diets

Study design

48 pigs divided in 5 groups (**Fig. 1**):
- 1 baseline group (BL, n = 8): BL was slaughtered after adaption period (3 weeks). All groups received the conventional diet during adaption period.
- 4 Feeding groups (feeding period of 15 weeks, **Tab. 1-2**)
  - Atherogenic diet (AD, n = 10)
  - Atherogenic diet + 5 % pectin (ADp, n = 10)
  - Atherogenic diet + 5 % inulin (ADi, n = 10)
  - Conventional diet (CD, n = 10) = control group

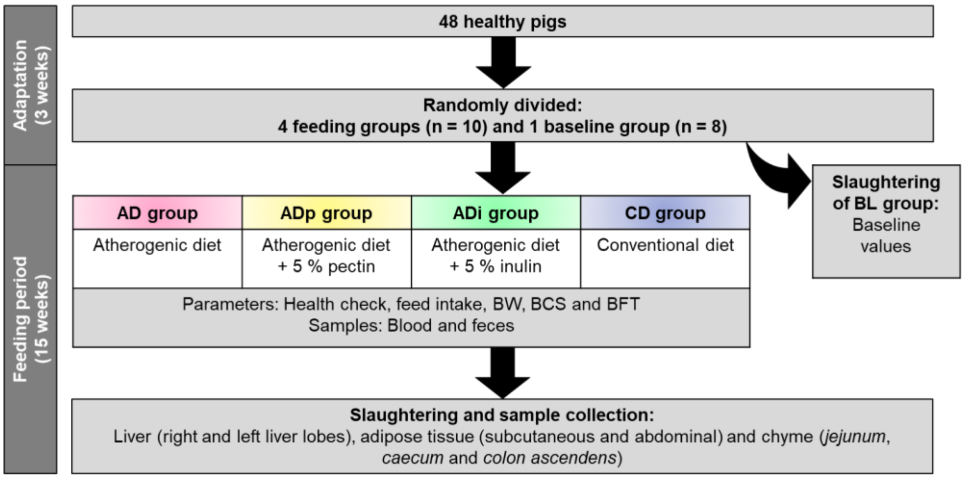

Fig 1. Study design. AD, group fed atherogenic diet (n = 10); ADp, group fed atherogenic diet + pectin (n = 10); ADi, group fed atherogenic diet + inulin (n = 10); CD, group fed conventional diet (n = 10); BL, baseline group; BW, body weight; BCS, body condition score; BFT, back fat thickness.

Tab. 1: Composition of atherogenic and conventional experimental diets

Feeding group	Experimental diet	
Atherogenic diet (AD)	10.0 %	Wheat
	18.0 %	Oats
	17.5 %	Soy bean meal
	2.50 %	Vitamins and Minerals[1]
	2.00 %	Fiber supplement[2]
	38.0 %	Crisps[3]
	10.0 %	Palm fat[4]

	2.00 %	Sugar
Atherogenic diet + 5 % pectin (ADp)	AD + 5 % pectin[5]	
Atherogenic diet + 5 % inulin (ADi)	AD + 5 % inulin[6]	
Conventional diet (CD)	43.5 %<br>38.0 %<br>14.0 %<br>2.50 %<br>2.00 %	Wheat<br>Oats<br>Soy bean meal<br>Vitamins and Minerals[1]<br>Fiber supplement[2]

Data are presented as component percentages. 1Troumix®M1, Trouw Nutrition Deutschland GmbH, Burgheim, Germany; 2FaserSpezial 2.0, Trouw Nutrition Deutschland GmbH; 3By-product of snack industry, feedstuff according to Regulation (EU) No. 575/2011; 4BEWI-SPRAY® 99L, BEWITAL agri GmbH & Co. KG, Südlohn-Oeding, Germany; 5agroPECT-A 100/70, agro Food Solution GmbH, Werder, Germany; 6Orafti® IPS; Beneo GmbH, Tienen, Belgium.

Tab. 2: Nutrients and calculated metabolizable energy levels of experimental diets

Crude nutrients in DM (%)	Feeding groups			
	AD	ADp	ADi	CD
CA	5.27	5.42	5.02	4.77
CL	27.2	25.7	25.2	3.40
CP	15.1	14.7	14.2	19.0
CF	7.22	7.94	7.34	7.13
NDF	11.7	10.7	10.7	21.7
ADF	5.60	5.56	5.45	9.46
NFE	39.2	39.9	41.9	55.4
Starch	34.4	34.3	32.4	45.5
Sugar	6.71	6.16	10.8	3.35
Sodium	0.34	0.35	0.34	0.14
**ME (MJ/kg DM)** [1]	**17.8**	**17.2**	**17.3**	**14.1**

The data are presented as percentages of DM. DM, dry matter; AD, atherogenic diet; ADp, atherogenic diet + 5 % pectin; ADi, atherogenic diet + 5 % inulin CD, conventional diet; CA, crude ash; CL, crude lipid; CP, crude protein; CF, crude fiber; NDF, ash-free neutral detergent fiber; ADF, ash-free acid detergent fiber; NFE, nitrogen-free extracts; ME, metabolizable energy.

[1] GfE. Communications of the Committee for Requirement Standards of the Society of Nutrition Physiology – prediction of metabolisable energy of compound feeds for pigs. Proceedings of the Society of Nutrition and Physiology. 2008;17: 199–204.

Slaughtering at the end of the 15-weeks feeding period for sample collection

- Stunning: electrical stunning equipment (TGB 200; Hubert Haas, Neuler, Germany)
- Blood withdrawal: severing brachiocephalic trunk and jugular vein
- Pigs were slaughtered in accordance with European and German law [Council Regulation (EC) No 1099/2009 of 24 September 2009, Tierschutz-Schlachtverordnung, § 4 Tierschutzgesetz].

Sample size

A total number of 48 pigs were included in this study. Pigs were divided into 5 groups:

- BL (n = 8)
- AD (n = 10)
- ADp (n = 10)
- ADi (n = 10)
- CD (n = 10)

Sample size calculation:

- Power analysis (power > 80 %, significance level at 5 %, SPSS Statistics 27.0, IBM, New York, USA) with the main target variable acetate as a function of carbohydrate intake (inulin, pectin) compared to the control group.
- Published data in pigs assume differences of acetate in the chyme of 200 mmol/kg dry mass in the chyme between the control group and the inulin / pectin fed animals. The standard variations varied between 50-62 mmol/kg dry mass in the chyme. The calculation of the required number of animals was

	between 8 and 9 pigs per group. We increase the number of animals to 10 per group to compensate for unexpected animal losses.
**Inclusion and exclusion criteria**	<u>Including criteria:</u><br>- Breed: Saddle back pigs (5 litters, same sire)<br>- Age: 5 months<br>- Health status: Healthy (examined by clinical examination and blood check)<br><u>Excluding criteria:</u><br>- A score sheet was developed to establish the criteria for excluding animals from the study. The score was developed specifically for pigs and modified based on the results for pain assessment in pigs by Ison et al. (2016).<br>- Scored parameter: general behaviour, condition score, feed and water intake, quality of faeces, breathing rate, body temperature, limbs, injuries (especially tail, ears and flanks), umbilical hernia, symptoms of gastric ulcers.<br>- Score > 0 to 1 is reached for one parameter → animals were intensively observed (clinical examination three times a day)<br>- Score > 1 is reached for more than one parameter or a score of 3 is reached for one parameter → the animals are excluded of the study and undergo further diagnostic examinations (e.g. further lameness diagnostics) and, if necessary, lege artis medical treatment (e.g. pain therapy or antibiosis).<br>- If a complete recovery of the animals is possible, they are included in the trial again.<br>- If individual animals cannot be recovered and included in the trial, these animals are euthanised to avoid further pain and suffering.
**Randomi-sation**	The pigs were randomly divided into five groups after weaning by the stable staff. These groups were maintained in the study to avoid rank fights between the animals. However, care was taken to ensure that animals from all litters and of all sexes were present in each group.
**Blinding**	The study was not blinded.
**Outcome measures**	<u>Outcome measures</u><br>- Feed intake<br>- Body weight (BW) development<br>- Body condition score (BCS) development<br>- Back fat thickness (BFT) development<br>- pH, dry matter (DM) and short chain fatty acids in feces and chyme<br>- Liver fat content<br>- Relative mRNA levels of inflammatory markers in liver and adipose tissue<br>- Number of macrophages in adipose tissue<br>The main target variable to determine the sample size was acetate in chyme as a function of carbohydrate intake (inulin, pectin) compared to the control group.
**Statistical methods**	Software<br>- SPSS Statistics version 27.0, IBM, Armonk, NY, USA<br>- Statistica 14.0, TIBCO, Palo Alto, CA, USA<br>Z-standardization<br>Check for normal distribution:<br>- Shapiro-Wilks Test (SPSS Statistics)<br>Logarithmically transformation, if the data sets were not normally distributed<br>Check for variance homogeneity:<br>- Levene Test (SPSS Statistics)<br>Normally distributed and homogenous data sets:<br>- BW, BCS, BFT (SPSS Statistics) propionate in feces, liver fat content, *IL–1β*, *IL–6*, *TNF–α*, *CD68* in liver (right liver lobe) and *IL–1β* (abdominal fat) in adipose tissue (Statistica)<br>➔ Repeated measures ANOVAs<br>➔ Post hoc test: Fisher's LSD test<br>(Tukey's HSD test for BW, BCS and BFT)<br>Normally distributed but not homogenous data sets:<br>- acetate in chyme and fecal pH (Statistica)<br>➔ Repeated measures ANOVAs<br>➔ Post hoc test: Games–Howell test<br>Not normally distributed data sets:

	- acetate in feces, propionate in chyme, total butyrate (= butyrate + isobutyrate), total valerate (= valerate + isovalerate), DM content in feces, *CD68* in liver (left liver lobe), *IL–1β* in subcutaneous fat, *IL–6*, *TNF–α*, *CD68* in adipose tissue and the number of macrophages in adipose tissue (SPSS Statistics)<br>➔ Kruskal-Wallis test with Bonferroni correction<br>Differences were considered significant for P values < 0.05. Data are shown as medians and [25th / 75th] percentiles for acetate, propionate, total butyrate and total valerate or mean values ± standard deviation (mean ± SD) for liver fat content and the number of macrophages in adipose tissue.
**Experimen-tal animals**	- 48 healthy, purebred saddleback pigs owned by the Institute of Animal Nutrition, Nutrition Diseases and Dietetics, Leipzig University<br>- female (n = 21) and castrated male pigs (n = 27) from five litters with the same sire<br>- pigs aged 5 months<br>- mean (± SD) body weight: 97.5 ± 9.36 kg.<br>- median and [25th / 75th] percentiles of body condition score (BCS): 3.13 [3.0 / 3.5] out of five<br>- mean (± SD) back-fat thickness (BFT): 20.3 ± 2.08 cm<br>- Pigs were housed in the same stable and separated in one pen per group according to the allotted treatment. The ambient temperature was 16–18 °C, and the humidity was 60–75 %. The pigs were bedded with wood shavings.<br>- Water was provided ad libitum by using an automatic watering system.<br>- Animals were adapted to the general experimental environment for at least 3 weeks. During the adaptation period, pigs were fed the same conventional diet.<br>- The project was approved by the Ethics Committee for Animal Rights Protection of the Leipzig District Government (no. TVV 04/20) in accordance with German legislation for animal rights and welfare.<br>- Two animal keepers provided the animals with food and cleaned the stable on a daily basis.<br>- Health status of the animals was examined by a Veterinarian in two-day intervals by clinical examination, including evaluations of general behavior, feed and water intake, fecal quality, breathing rate, and body temperature. In addition, blood tests (blood count and chemistry) were performed before and after the feeding period.<br>- Temperature and humidity in the stable were recorded daily.
**Experimen-tal procedu-res**	<u>Morphometric measurements</u><br>Body weight was measured weekly with a portable electronic scaling system (Minipond 21, Baumann Waagen und Maschinenbau GmbH, Thiersheim, Germany). BCS was evaluated weekly using a scale from 0 to 5 [Young et al. 2001]. Monthly, BFT was obtained by transcutaneous ultrasound measurements (Portable Ultrasonic Diagnostic System A6V, SonoScape Co., Shenzhen, China) of six measuring points according to the ABC-6-methode [Spanlang 2011].<br><u>Blood sampling</u><br>Blood samples were collected at the beginning of the study (t0; October, 8 to October,13, 2020). Blood was taken by single puncture of the left or right jugular vein. Follow up blood samples of the four feeding groups were sampled after one (t1; November, 11, 2020), two (t2; December, 17, 2020) and three months (t3; January, 21, 2021) of feeding the experimental diets. For blood chemistry and metabolome analyses serum tubes (Monovette® Z, Sarstedt AG & Co. KG, Nümbrecht, Germany) containing coagulation activator were used. For blood count tubes containing EDTA (Monovette® K3E (1.6 mg EDTA/mL), Sarstedt AG & Co. KG) were taken and immediately analysed after sampling. Serum tubes were centrifuged after 30 min of clotting at room temperature and then frozen in multiple aliquots of 1mL at −80°C until analysis.<br><u>Faeces sampling</u><br>Faecal samples were collected at the same time points (t0; t1; t2; t3) as the blood samples. Rectal faeces were collected from each animal.<br><u>Slaughtering and sampling of liver, adipose tissue and chyme</u><br>The pigs were stunned with an electrical stunning equipment. Blood withdrawal followed immediately after electrical stunning by severing brachiocephalic trunk and

jugular vein. After slaughter, half of the *lobus hepatis sinister lateralis* and *lobus hepatis dexter lateralis* and the gastrointestinal tract in toto were removed for subsequent sampling. Immediately, 5 g of each liver lobe were obtained aseptically, shock-frozen in liquid nitrogen and stored at -80°C until analysis of inflammatory markers. Adipose tissue was collected from two localizations. Subcutaneous adipose tissue was harvested from the back fat located above the 7th cervical vertebra and abdominal adipose tissue from the area of the mesocolon ascendens. As described for the liver, 5 g of adipose tissue samples were immediately shock-frozen. Two other pieces of 1 cm3 were fixed in 4 % neutral-buffered formaldehyde (OMEGA Pharma Deutschland, Herrenberg, Germany) for at least two days to prepare polyethylene glycol sections as well as for immunohistochemical examination. After the adipose tissue was removed, chyme was taken from the middle of jejunum, caecum, and colon ascendens. Samples in duplicates of 13 g were collected in tubes from each of the three intestinal locations. Chyme pH was determined immediately by pH meter (PHM 93 Reference pH meter, Radiometer Copenhagen) and DM content of chyme was determined after oven-drying (103°C). Chyme samples were frozen at -80°C for further analysis of SCFA.

Results

Health monitoring

All pigs in the groups ADi and CD were clinically healthy during the 15-week feeding period. In the AD group, three pigs (one pig twice) were treated with nonsteroidal anti-inflammatory drugs (Melosolute: 20 mg/mL meloxicam; CP-Pharma, Burgdorf, Germany) for five to seven days during the 15-week feeding period because of lameness resulting from joint inflammation. Two of the animals also developed fever and were additionally treated with antibiotics (Hostamox LA: 150 mg/mL amoxicillin; MSD, Rahway, NJ, USA) for three days. In the ADp group, two animals were treated with nonsteroidal anti-inflammatory drugs for three days each. One of the pigs was moderately lame due to an inflamed bursa, and the other pig was lame as a result of slipping on the stable gangway. To avoid any interference with the microflora or inflammatory response in the liver and adipose tissue, an interval of at least two weeks was maintained between the treatment and the collection of samples. All of the sick pigs were successfully treated and did not need to be excluded from the experiment at any of the sampling time points. **Tab. 3** shows the increases in BW, BCS and BFT. The results of the statistical analysis of these parameters are presented in Wahl et al. (2022) [52].

Tab. 3: BW, BCS, and BFT development in the four feeding groups during the treatment period

Parameter	Groups	Start of feeding period	End of feeding period	Increase (%)
BW (kg) P = 0.82	AD	110 ± 12.2	177 ± 14.1	61.2 ± 16.3
	ADp	111 ± 6.51	176 ± 12.4	58.1 ± 12.0
	ADi	113 ± 11.7	183 ± 16.2	62.1 ± 11.8
	CD	102 ± 4.69	162 ± 6.00	58.9 ± 8.60
BCS (scale 1–5) P = 0.99	AD	3.55 ± 0.37	4.65 ± 0.15	32.1 ± 13.9
	ADp	3.43 ± 0.17	4.58 ± 0.11	33.8 ± 6.30
	ADi	3.53 ± 0.34	4.68 ± 0.20	33.6 ± 11.2
	CD	3.30 ± 0.25	4.36 ± 0.11	33.0 ± 12.3
BFT (mm) P = 0.42	AD	19.7 ± 4.27	40.8 ± 5.26	110 ± 41.9
	ADp	20.4 ± 2.05	38.0 ± 6.12	90.9 ± 45.3
	ADi	21.6 ± 3.38	43.8 ± 7.14	109 ± 53.1
	CD	19.0 ± 5.19	32.7 ± 3.79	82.3 ± 44.7

Data are presented as mean ± SD. Significant differences between feeding groups (n = 10) were identified by P values ≤ 0.05, using repeated measures ANOVA with Tukey's HSD. AD, group fed atherogenic diet; ADp, group fed atherogenic diet + pectin; ADi, group fed atherogenic diet + inulin; CD, group fed conventional diet; BW, body weight; BCS, body condition score; BFT, back fat thickness. The observation period was 15 weeks.

Lipid parameters in serum

The serum parameters triglycerides (TG; t1 and t2: P ≤ 0.01), bile acids (BA; t1 and t2: P ≤ 0.02) and hepatic triglyceride lipase (LIPC; t1: P ≤ 0.02) presented significantly

greater values in the atherogenic diet groups than in the CD group. A detailed evaluation of the serum parameters of liver and lipid metabolism [TG, BA, LIPC, cholesterol (CHOL), alkaline phosphatase (ALP), aspartate aminotransferase (AST), gamma-glutamyl transferase (GGT), lactate dehydrogenase (LDH) and amylase (AMYL)] is described in Wahl et al. (2022).

Fecal pH and DM

An increase in the faecal pH and dry matter (DM) content was found in all the groups from t0 to t3 (**Tab. 4**). There were no significant differences in pH and DM content between the AD and CD group at any time (t0–t3). At t1, the pH was markedly higher in the CD group than in the ADp and ADi groups (P = 0.01), without significant differences between AD, ADp and ADi. At t2, the DM content of the AD group was significantly lower than that of the ADp group (P = 0.05).

Tab. 4: pH, DM content and SCFA concentrations in faeces before the feed change (t0) and at 1–3 months after the feed change (t1–t3).

SCFA	Groups	t0	t1	t2	t3
**pH**	AD	6.64$^{\#}$<br>[6.46/6.88]	6.94$^{\#▲, ab}$<br>[6.59/7.13]	7.02$^{\#▲}$<br>[6.90/7.10]	7.16$^{▲}$<br>[6.91/7.70]
	ADp	6.58$^{\#}$<br>[6.47/6.67]	6.60$^{\#, a}$<br>[6.51/6.84]	6.91$^{▲}$<br>[6.80/6.99]	6.84$^{\#▲}$<br>[6.48/7.14]
	ADi	6.64$^{\#}$<br>[6.59/6.68]	6.63$^{\#, a}$<br>[6.37/6.73]	7.03$^{▲}$<br>[6.84/7.19]	6.84$^{\#▲}$<br>[6.67/7.25]
	CD	6.65$^{\#}$<br>[6.51/6.71]	6.93$^{\#, b}$<br>[6.80/7.13]	7.06$^{▲}$<br>[6.89/7.24]	6.95$^{\#▲}$<br>[6.61/7.52]
**DM**	AD	21.6$^{\#}$<br>[20.8/22.1]	27.4$^{▲}$<br>[25.6/28.7]	25.7$^{▲, a}$<br>[24.2/28.1]	31.2$^{▲}$<br>[28.3/35.0]
	ADp	21.5$^{\#}$<br>[20.4/22.5]	24.9$^{\#▲}$<br>[22.7/26.5]	31.5$^{■, b}$<br>[28.9/33.6]	30.1$^{▲■}$<br>[27.6/32.0]
	ADi	20.9$^{\#}$<br>[20.0/21.5]	26.1$^{▲}$<br>[24.3/29.6]	26.9$^{▲, ab}$<br>[25.0/30.6]	28.7$^{▲}$<br>[24.9/32.2]
	CD	21.2$^{\#}$<br>[20.7/22.4]	25.5$^{▲}$<br>[23.9/26.2]	25.9$^{▲, ab}$<br>[25.0/30.1]	26.2$^{▲}$<br>[24.8/30.3]
**Acetate**	AD	98.9$^{\#}$<br>[80.3/105]	82.7$^{\#▲, ab}$<br>[77.1/95.5]	76.4$^{\#▲}$<br>[73.5/84.1]	73.0$^{▲}$<br>[66.1/96.0]
	ADp	100$^{\#}$<br>[89.4/103]	82.2$^{▲, a}$<br>[76.2/84.3]	84.6$^{▲}$<br>[67.9/92.0]	84.3$^{\#▲}$<br>[75.6/95.4]
	ADi	98.9<br>[89.0/105]	91.5^{ab}<br>[81.0/104]	91.4<br>[77.8/106]	88.7<br>[83.7/99.5]
	CD	88.1<br>[81.0/99.2]	96.9^{b}<br>[91.1/107]	95.5<br>[74.5/104]	94.5<br>[86.4/104]
**Propionate**	AD	41.6$^{\#, a}$<br>[32.7/45.4]	27.9$^{▲}$<br>[24.3/33.2]	23.8$^{▲}$<br>[21.1/27.6]	23.4$^{▲}$<br>[20.8/28.5]
	ADp	36.9$^{\#, ab}$<br>[34.4/42.7]	26.1$^{▲}$<br>[24.5/27.7]	26.8$^{▲}$<br>[22.3/30.5]	24.2$^{▲}$<br>[21.1/27.9]
	ADi	33.5$^{\#, ab}$<br>[31.8/35.5]	28.4$^{▲}$<br>[24.8/32.6]	27.9$^{▲}$<br>[23.5/30.3]	23.6$^{▲}$<br>[19.3/29.7]
	CD	33.5$^{\#, b}$<br>[29.2/37.0]	31.0$^{\#}$<br>[27.7/36.0]	28.6$^{▲}$<br>[21.8/30.1]	30.4$^{\#▲}$<br>[26.0/32.8]
**Total butyrate**	AD	29.9$^{\#}$<br>[26.8/38.8]	16.9$^{▲}$<br>[14.5/21.4]	14.4$^{▲}$<br>[8.94/17.1]	14.2$^{▲}$<br>[12.2/16.3]
	ADp	36.3$^{\#}$<br>[28.7/39.6]	17.0$^{▲}$<br>[14.0/18.1]	17.1$^{▲}$<br>[13.6/19.2]	14.6$^{▲}$<br>[11.0/17.2]
	ADi	30.3$^{\#}$<br>[26.3/31.9]	20.9$^{▲}$<br>[15.8/22.2]	17.9$^{▲}$<br>[15.2/21.8]	15.2$^{▲}$<br>[13.7/18.1]
	CD	30.3$^{\#}$<br>[21.7/33.9]	19.8$^{\#▲}$<br>[17.7/22.8]	19.4$^{▲}$<br>[11.9/21.2]	18.5$^{▲}$<br>[16.3/24.3]
**Total valerate**	AD	9.65$^{\#}$<br>[7.06/11.8]	7.53$^{\#, ab}$<br>[6.62/8.52]	5.56$^{▲}$<br>[5.18/6.59]	6.84$^{\#▲}$<br>[5.79/7.42]
	ADp	8.24$^{\#}$<br>[7.11/9.35]	6.36$^{▲, a}$<br>[5.44/7.04]	6.70$^{\#▲}$<br>[5.83/8.16]	6.66$^{\#▲}$<br>[6.34/7.13]
	ADi	6.91<br>[5.97/9.30]	7.68^{ab}<br>[6.88/8.36]	7.47<br>[6.19/8.37]	6.95<br>[4.98/8.87]
	CD	8.40<br>[6.83/10.6]	7.77^{b}<br>[7.28/8.88]	6.96<br>[6.01/7.95]	7.97<br>[7.34/8.34]

The SCFA concentrations in faeces of all feeding groups (AD, ADp, ADi and CD) before the feed change (t0) and at 1–3 months after the feed change (t1–t3) are presented as medians and [25th/75th] percentiles in mmol/kg DM. $^{▲\#■}$Different symbols indicate significant differences within a row (time point differences in one

group). [ab]Different lowercase letters indicate significant effects within a column (group differences at one time point). Significant differences are identified by P values ≤ 0.05 via repeated-measures ANOVA with Fisher´s LSD test (propionate) or with the Games–Howell test (faecal pH) and the Kruskal–Wallis test with Bonferroni correction (DM content, acetate, total butyrate and total valerate). SCFA, short-chain fatty acid; DM, dry matter; BL, baseline group (n = 8); AD, group fed the atherogenic diet (n = 8–10); ADp, group fed the atherogenic diet + pectin (n = 9–10); ADi, group fed the atherogenic diet + inulin (n = 9–10); CD, group fed the conventional diet (n = 9–10); n/a, not available.

<u>SCFA in feces and chyme</u>
The SCFA concentration in faeces decreased over time from t0–t3 in all groups (**Tab. 4**). A decrease was particularly observed in the AD, ADp and ADi groups, in which the feed was changed to an atherogenic diet after t0. No significant differences in SCFAs such as acetate, propionate, total butyrate and total valerate were found between the AD and CD groups at any time point (t0–t3), except before feed change (t0) with higher propionate levels in the AD group (P = 0.04). There were also no significant differences in SCFA levels between the AD, ADp and ADi groups at any time point (t0–t3). At t1, the faecal acetate and valerate levels were significantly lower in the ADp group than in the CD group (P ≤ 0.04).
When the SCFA concentrations were compared between the jejunum, caecum, ascending colon and time point t3 faecal samples, the acetate, propionate, total butyrate and total valerate levels were significantly lower in the jejunum than in the caecum, colon and faeces (P ≤ 0.005; **Fig. 2** and **Tab. 5**). The highest concentrations of acetate and propionate were present in the caecum. The SCFA levels in the colon and faeces were in a similar range. No significant differences in total butyrate and valerate were found between the caecum, colon and faeces. Except for faeces, the BL group showed higher concentrations of SCFAs in chyme at all locations than the four feeding groups (AD, ADp, ADi and CD).No significant differences were found between the AD and CD groups or between the AD, ADp and ADi groups in SCFA levels or intestinal location.

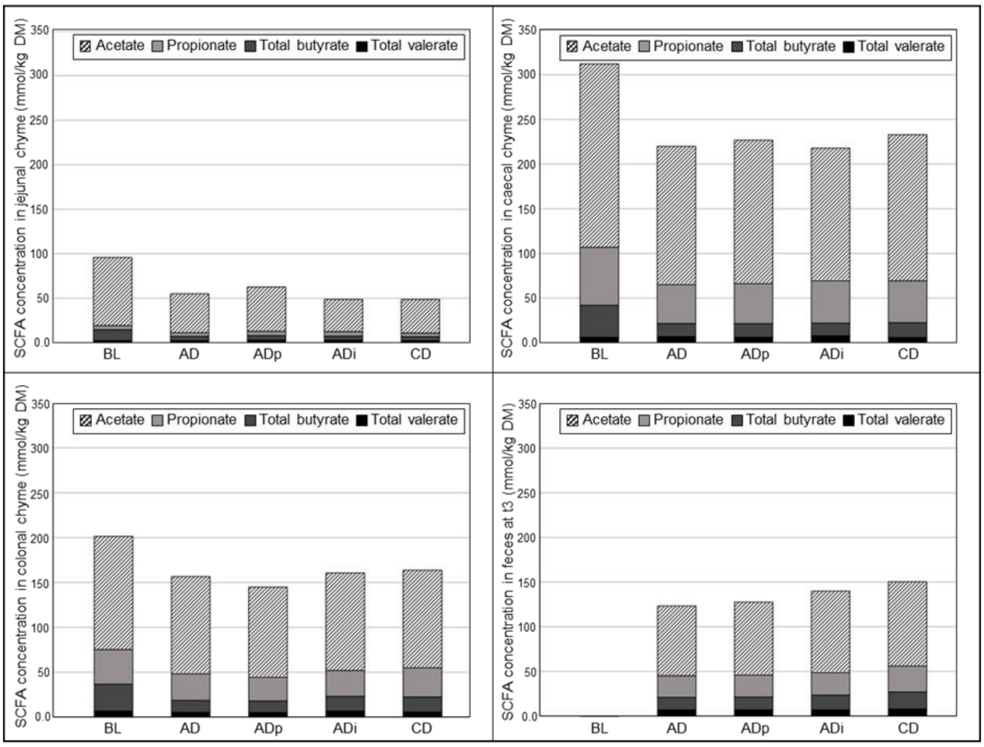

Fig 2. SCFA concentrations (mmol/kg DM) in chyme and feces. Data present the SCFA concentration in jejunum, caecum and colon at time of slaughter and in feces at time t3 for all groups (BL, AD, ADp, ADi, CD). SCFA, short chain fatty acids; DM, dry matter; BL, baseline group (n = 6–8); AD, group fed atherogenic diet (n = 9–10); ADp,

	group fed atherogenic diet + pectin (n = 9–10); ADi, group fed atherogenic diet + inulin (n = 9–10); CD, group fed conventional diet (n = 9–10).<br><br>**Tab. 5**: SCFA concentration in chyme of several intestinal locations and feces.

SCFA	Groups	*Jejunum*	*Caecum*	*Colon*	Feces (t3)
**Acetate**	BL	76.5[a]<br>[67.2 / 83.6]	208<br>[179 / 234]	136<br>[119 / 139]	n/a
	AD	36.4[#, ab]<br>[36.4 / 76.1]	123[▲]<br>[101 / 225]	101[■▲]<br>[76.2 / 150]	73.0[#]<br>[66.1 / 96.0]
	ADp	46.9[#, ab]<br>[26.3 / 65.4]	142[▲]<br>[120 / 201]	95.3[■]<br>[79.5 / 129]	95.3[■]<br>[79.5 / 129]
	ADi	33.0[#, b]<br>[27.2 / 40.6]	163[▲]<br>[138 / 177]	107[▲■]<br>[87.9 / 132]	88.7[■]<br>[83.7 / 99.5]
	CD	39.5[#, b]<br>[25.4 / 47.8]	178[▲]<br>[132 / 206]	111[■]<br>[81.7 / 126]	94.5[■]<br>[86.4 / 104]
**Propionate**	BL	4.54<br>[4.03 / 4.86]	66.9<br>[48.6 / 77.4]	41.8[a]<br>[34.5 / 45.7]	n/a
	AD	4.13[#]<br>[3.73 / 5.24]	35.8[▲]<br>[29.5 / 60.8]	30.2[▲, ab]<br>[26.0 / 35.8]	23.4[#▲]<br>[20.8 / 28.5]
	ADp	4.87[#]<br>[3.99 / 6.10]	46.1[▲]<br>[35.0 / 59.3]	25.0[▲■, b]<br>[21.7 / 32.0]	24.2[#■]<br>[21.1 / 27.9]
	ADi	4.64[#]<br>[3.75 / 6.20]	48.2[▲]<br>[42.3 / 61.0]	27.5[▲■, ab]<br>[24.9 / 35.1]	23.6[#■]<br>[19.3 / 29.7]
	CD	3.35[#]<br>[2.82 / 5.71]	49.5[▲]<br>[38.0 / 59.6]	31.8[▲, ab]<br>[26.6 / 38.0]	30.4[▲]<br>[26.0 / 32.8]
**Total butyrate**	BL	11.8[a]<br>[9.84 / 14.4]	35.8[a]<br>[33.8 / 38.5]	29.2[a]<br>[27.0 / 37.4]	n/a
	AD	4.45[#, b]<br>[4.23 / 5.64]	12.9[▲, b]<br>[9.65 / 21.5]	12.6[▲, b]<br>[11.3 / 15.4]	14.2[▲]<br>[12.2 / 16.3]
	ADp	4.86[#, ab]<br>[4.21 / 5.75]	12.3[▲, b]<br>[10.8 / 20.0]	11.3[▲, b]<br>[10.8 / 15.0]	14.6[▲]<br>[11.0 / 17.2]
	ADi	4.05[#, b]<br>[3.43 / 5.03]	14.5[▲, b]<br>[13.3 / 15.9]	15.8[▲, ab]<br>[14.3 / 18.1]	15.2[▲]<br>[13.7 / 18.1]
	CD	4.20[#, b]<br>[2.62 / 5.69]	17.1[▲, b]<br>[11.8 / 20.7]	16.4[▲, ab]<br>[12.1 / 21.2]	18.5[▲]<br>[16.3 / 24.3]
**Totel valeriate**	BL	2.79<br>[2.30 / 3.74]	5.92<br>[5.61 / 7.07]	6.82[a]<br>[5.91 / 7.54]	n/a
	AD	2.24[#]<br>[1.96 / 2.74]	6.13[▲]<br>[5.29 / 8.73]	5.67[▲, ab]<br>[4.44 / 5.98]	6.84[▲]<br>[5.79 / 7.42]
	ADp	3.04[#]<br>[2.36 / 3.25]	6.08[▲]<br>[5.12 / 7.27]	5.35[#▲, b]<br>[4.23 / 5.87]	6.66[▲]<br>[6.34 / 7.13]
	ADi	2.67[#]<br>[2.15 / 3.32]	7.87[▲]<br>[6.34 / 10.6]	6.33[▲, ab]<br>[5.16 / 7.83]	6.95[▲]<br>[4.98 / 8.87]
	CD	1.81[#]<br>[1.39 / 2.99]	5.34[▲]<br>[4.95 / 6.76]	5.82[▲, ab]<br>[4.52 / 6.06]	7.97[▲]<br>[7.34 / 8.34]

Data present the SCFA concentration in jejunum, caecum and colon at time of slaughter and in feces at time t3 for all groups (BL, AD, ADp, ADi, CD) and are presented as medians and [25th / 75th] percentiles in mmol/kg DM. [▲#■]Different symbols indicate significant differences within a row (intestinal section differences in one group). [ab]Different lowercase letters indicate significant effects within a column (group differences at one intestinal section). Significant differences are identified by P values ≤ 0.05 using repeated measures ANOVA with Games–Howell test (acetate) and Kruskal–Wallis test with Bonferroni correction (propionate, total butyrate and total valerate). SCFA, short chain fatty acids; DM, dry matter; BL, baseline group (n = 6–8); AD, group fed atherogenic diet (n = 9–10); ADp, group fed atherogenic diet + pectin (n = 9–10); ADi, group fed atherogenic diet + inulin (n = 9–10); CD, group fed conventional diet (n = 9–10); n/a, not available.

<u>Liver fat content</u>

The crude lipid (CL) content of liver tissue (Table 3) was within the physiological range for pig liver [46] and was similar between the BL group and the feeding groups (AD, ADp, ADi and CD; **Tab. 6**). Between AD and CD groups no significant difference was found for CL. Focusing on the AD, ADp and ADi groups, a significant difference was determined only in the *lobus hepatis dexter lateralis* between ADp and ADi groups with lower values in ADi group (P = 0.002). There was also a significant difference in the *lobus hepatis dexter lateralis* between the BL group and the AD, ADp, and CD groups (P = 0.002).

Tab. 6: Crude lipid content in liver tissue of the BL group and of each dietary group (% in DM)

Liver tissue	BL	AD	ADp	ADi	CD
Left liver lobe	9.62 ± 1.12	9.67 ± 0.81	10.1 ± 0.99	10.5 ± 1.13	10.3 ± 1.70
Right liver lobe	8.89 ± 1.17 ^{a}	10.6 ± 0.60^{bc}	10.9 ± 1.88^{c}	9.60 ± 0.80^{ab}	11.1 ± 1.15^{c}

Data are presented as means ± SD in percentages of DM. ^{abc}Different lowercase letters indicate significant effects between the groups, identified by P values ≤ 0.05, using ANOVA with Fisher's LSD test. Left liver lobe, *lobus hepatis sinister lateralis*; Right liver lobe, *lobus hepatis dexter lateralis*; CL, crude lipid; DM, dry matter; BL, baseline group (n = 8); AD, group fed an atherogenic diet (n = 10); ADp, group fed an atherogenic diet + pectin (n = 10); ADi, group fed an atherogenic diet + inulin (n = 10); CD, group fed a conventional diet (n = 10).

<u>Inflammatory markers in liver and adipose tissue</u>

In the liver, the relative mRNA levels differed between the *lobus hepatis sinister lateralis* and the *lobus hepatis dexter lateralis* (**Fig. 3**). The relative mRNA levels of *IL–1β* and *IL–6* in the liver were on average < 1 and did not significantly differ between the groups. For *TNF–α*, the relative mRNA levels in the right liver lobe, but not in the left lobe, differed significantly between the groups (P = 0.007). Relative mRNA levels of *TNF–α* in the right liver lobe were significant lower in AD, than in CD group (P = 0.001). Between the groups, in which the diet changed to an atherogenic diet after t0, significantly lower *TNF–α* levels were found in the AD group compared to the ADi group (P = 0.01). The relative *TNF–α* mRNA levels in the left liver lobe were on average < 1 and did not differ between the groups. The mean *cluster of differentiation 68* (*CD68*) mRNA level in the left liver lobe exceeded on average 1 in the BL group and the ADp group, whereas all the levels in the right liver lobe were on average < 1. No significant effects were found for *CD68* mRNA levels in the liver.

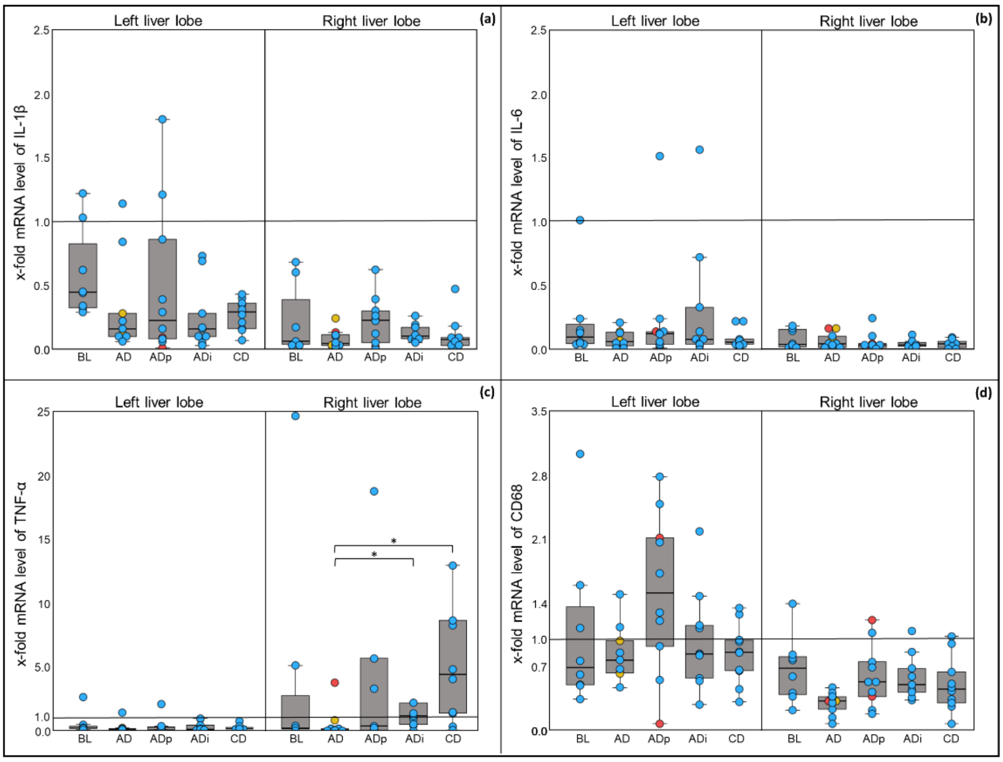

Fig 3. mRNA levels of *IL–1β, IL–6, TNF–α* and *CD68* in the liver tissue. The data are presented as box plots with a single dot per animal. Animals treated with drugs are marked with red dots for nonsteroidal anti-inflammatory drugs and yellow dots for antibiotics. *Asterisks indicate significant differences between the groups. Significant differences were identified by P values ≤ 0.05 via ANOVA with Fisher's LSD test for

IL–1β, IL–6, TNF–α and *CD68* (right liver lobe) and the Kruskal–Wallis test with Bonferroni correction for *CD68* (left liver lobe). Left liver lobe, *lobus hepatis sinister lateralis*; Right liver lobe, *lobus hepatis dexter lateralis*; IL–1β, interleukin–1β; IL–6, interleukin–6; TNF–α, tumour necrosis factor α; CD68, cluster of differentiation 68; BL, baseline group (n = 7–8); AD, group fed the atherogenic diet (n = 8–10); ADp, group fed the atherogenic diet + pectin (n = 9–10); ADi, group fed the atherogenic diet + inulin (n = 9–10); CD, group fed the conventional diet (n = 10).

The relative mRNA levels of inflammatory markers in the adipose tissue differed between subcutaneous and abdominal adipose tissue, with considerably higher levels in the abdominal adipose tissue, excluding *CD68* in the AD and ADp groups (**Fig. 4**). Inflammatory marker expression was greater in the abdominal adipose tissue than in the subcutaneous adipose tissue, as follows: *IL–1β*: 1.36 to 8.09-fold, *IL–6*: 2.20 to 15.7-fold and *TNF–α*: 10.8 to 433-fold. The levels of *CD68* in the BL, ADi and CD groups were 2.45 to 3.33 times higher in the abdominal adipose tissue than they were in the subcutaneous adipose tissue, whereas in the AD and ADp groups, the *CD68* levels were half as high in the abdominal adipose tissue as they were in the subcutaneous adipose tissue.
In the abdominal adipose tissue the *IL–1β* mRNA levels were significantly lower in AD than in CD group (P = 0.03). In the groups fed an atherogenic diet, the *IL–1β* mRNA levels were significant higher in ADi group than in AD and ADp groups (P = 0.02). Similar results were found for the mRNA levels of *CD68* in the abdominal adipose tissue, with significantly lower *CD68* mRNA levels in the AD group than in the CD group (P = 0.02). Comparing the groups fed an atherogenic diet, *IL-1β* mRNA levels were significantly higher in the ADi group than in the ADp group (P = 0.03). The mRNA levels of *IL–6* in the abdominal adipose tissue showed no significant differences between AD and CD groups, but tended to be higher in the ADi group compared to AD and ADp groups (P = 0.087). The *TNF–α* mRNA levels in abdominal adipose tissue were numerically higher in the CD group than in the AD group (P = 0.087), but no significant differences were found between the groups fed an atherogenic diet.
In the subcutaneous adipose tissue, no significant differences were observed between AD and CD group or between the groups fed the atherogenic diet. A significantly lower *IL-6* mRNA level was found in the BL group compared to ADi (P = 0.02). Overall, a large animal-individual variation was observed for inflammatory mRNA expression in the different tissues, namely in the adipose tissue.

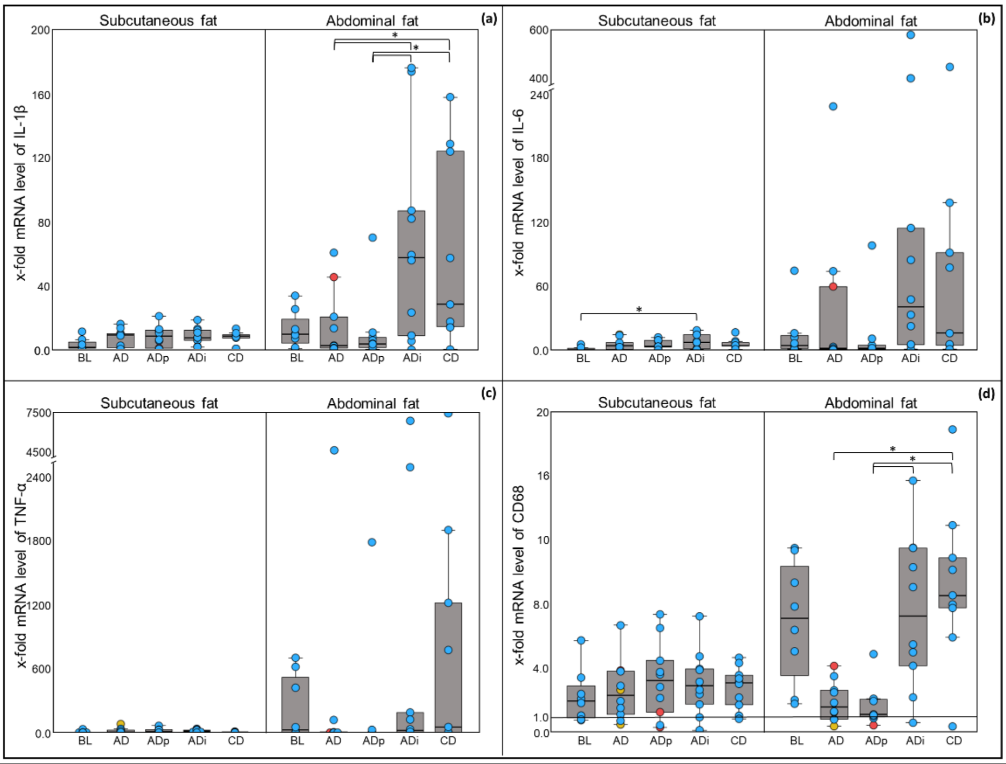

Fig 4. mRNA levels of *IL–1β, IL–6, TNF–α* and *CD68* in adipose tissue. The data are presented as box plots with a single dot per animal. Animals treated with drugs are marked with red dots for nonsteroidal anti-inflammatory drugs and yellow dots for antibiotics. *Asterisks indicate significant differences between the groups. Significant differences were identified by P values ≤ 0.05 via ANOVA with Fisher's LSD for *IL–1β* (abdominal fat) and the Kruskal–Wallis test with Bonferroni correction for *IL–1β* (subcutaneous fat), *IL–6, TNF–α*, and *CD68*. IL–1β, interleukin–1β; IL–6, interleukin–6; TNF–α, tumour necrosis factor α; CD68, cluster of differentiation 68; BL, baseline group (n = 8); AD, group fed the atherogenic diet (n = 10); ADp, group fed the atherogenic diet + pectin (n = 10); ADi, group fed the atherogenic diet + inulin (n = 9–10); CD, group fed the conventional diet (n = 9–10).

Number of macrophages in adipose tissue

The number of Iba-1-positive macrophages per mm^2 (**Fig. 5**) in the abdominal and subcutaneous adipose tissue did not significantly differ between the groups (P = 0.3). There was only a trend in the subcutaneous adipose tissue to a greater number of macrophages in the AD and ADp groups than in the BL group (P = 0.09; **Tab. 7**).

Tab. 7: Number of macrophages in adipose tissue (mean number/mm^2)

Adipose tissue	BL	AD	ADp	ADi	CD
Subcutaneous fat	4.13 ± 2.36	9.64 ± 5.13	7.54 ± 3.61	5.84 ± 3.29	5.35 ± 2.71
Abdominal fat	6.21 ± 2.31	7.40 ± 4.00	7.31 ± 2.96	5.78 ± 1.93	5.24 ± 4.24

The data are presented as the means ± SDs of the number of macrophages/mm^2. Significant differences were identified by P values ≤ 0.05 via Kruskal–Wallis test with Bonferroni correction. BL, baseline group (n = 8); AD, group fed the atherogenic diet (n = 10); ADp, group fed the atherogenic diet + pectin (n = 10); ADi, group fed the atherogenic diet + inulin (n = 10); CD, group fed the conventional diet (n = 10).

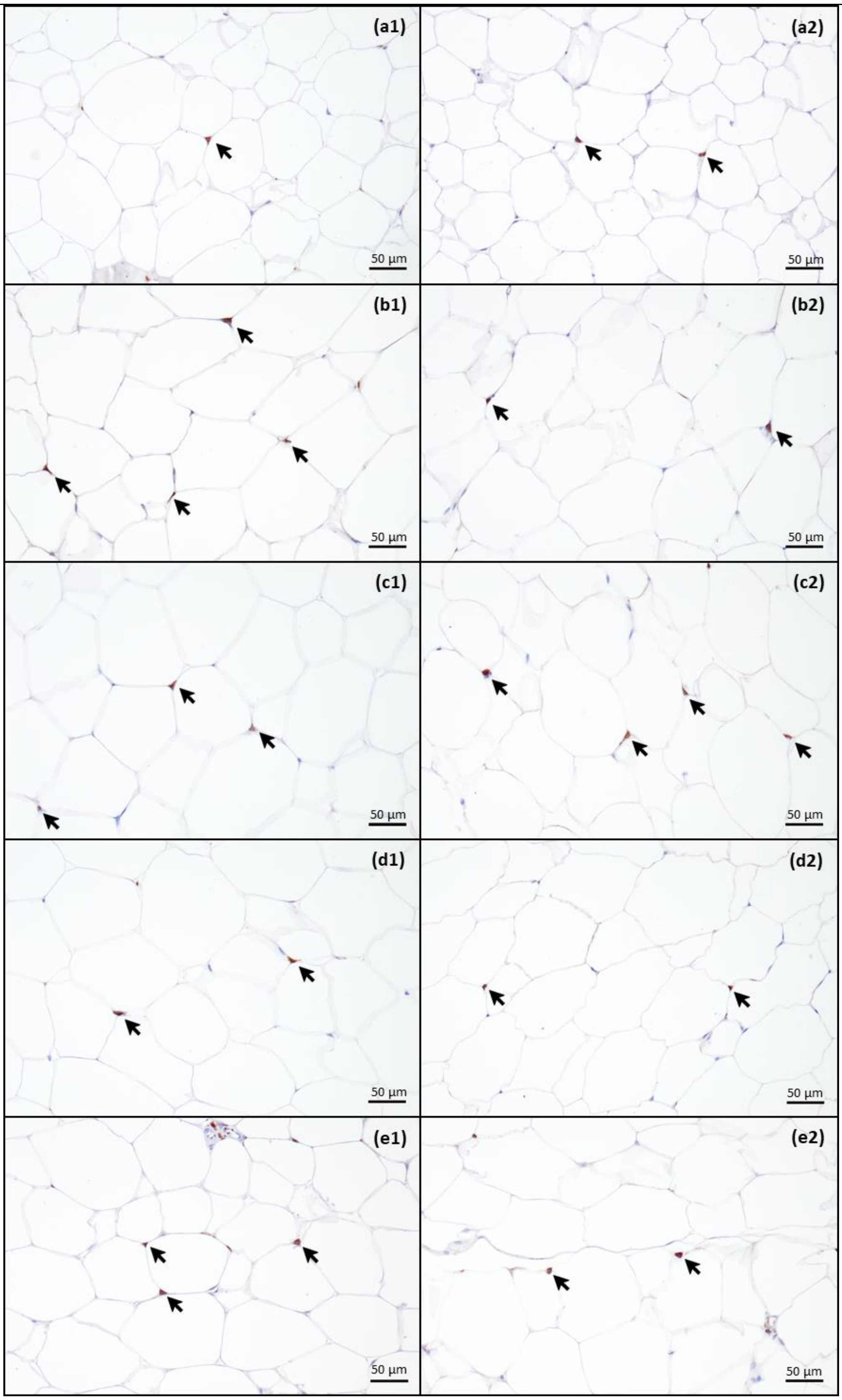

Fig 5. Iba-1-positive macrophages in adipose tissue. Presented are two images per group [BL group (a); AD group (b); ADp group (c); Adi group (d); CD group (e)] of the subcutaneous (1) and abdominal adipose tissue (2) with 20x magnification. Arrows mark brown-stained macrophages. BL, baseline group; AD, group fed the atherogenic diet; ADp, group fed the atherogenic diet + pectin; ADi, group fed the atherogenic diet + inulin; CD, group fed the conventional diet.
